# A systematic review and meta‐analysis of gene therapy in animal models of cerebral glioma: why did promise not translate to human therapy?

**DOI:** 10.1002/ebm2.6

**Published:** 2015-01-20

**Authors:** T. C. Hirst, H. M. Vesterinen, S. Conlin, K. J. Egan, A. Antonic, A. Lawson McLean, M. R. Macleod, R. Grant, P. M. Brennan, E. S. Sena, I. R. Whittle

**Affiliations:** ^1^ Centre for Clinical Brain SciencesChancellors BuildingUniversity of EdinburghEdinburghUK; ^2^ Florey Neuroscience and Mental Health InstituteUniversity of MelbourneVictoriaAustralia; ^3^ Division of Clinical NeurosciencesUniversity of EdinburghWestern General HospitalEdinburghUK

**Keywords:** systematic review, rodent models, glioma, gene therapy

## Abstract

**Background:**

The development of therapeutics is often characterized by promising animal research that fails to translate into clinical efficacy; this holds for the development of gene therapy in glioma. We tested the hypothesis that this is because of limitations in the internal and external validity of studies reporting the use of gene therapy in experimental glioma.

**Method:**

We systematically identified studies testing gene therapy in rodent glioma models by searching three online databases. The number of animals treated and median survival were extracted and studies graded using a quality checklist. We calculated median survival ratios and used random effects meta‐analysis to estimate efficacy. We explored effects of study design and quality and searched for evidence of publication bias.

**Results:**

We identified 193 publications using gene therapy in experimental glioma, including 6,366 animals. Overall, gene therapy improved median survival by a factor of 1.60 (95% CI 1.53–1.67). Study quality was low and the type of gene therapy did not account for differences in outcome. Study design characteristics accounted for a significant proportion of between‐study heterogeneity. We observed similar findings in a data subset limited to the most common gene therapy.

**Conclusion:**

As the dysregulation of key molecular pathways is characteristic of gliomas, gene therapy remains a promising treatment for glioma. Nevertheless, we have identified areas for improvement in conduct and reporting of studies, and we provide a basis for sample size calculations. Further work should focus on genes of interest in paradigms recapitulating human disease. This might improve the translation of such therapies into the clinic.

## Introduction

The prognosis for patients with malignant glioma remains poor despite extensive experimental and clinical research.^1^ The most effective treatments tested in randomized controlled trials show a median survival of 14 months from diagnosis,^2^ only marginal progress from the 9 months median survival reported in clinical trials in the 1970s.^3^, ^4^ This poor prognosis reflects the highly invasive nature of malignant glioma and resistance to conventional anticancer treatments.^5^, ^6^, ^7^ These tumours are proliferating lesions within an otherwise quiescent organ and rarely metastasize, rather progressing by diffuse invasion along white matter tracts and by inducing oedema through generation of abnormal blood vessels.^8^ Therefore they are an ideal target for local gene therapy, as such genes can be designed to target mitotic cells,^9^, ^10^ providing a basis for cell‐selective cytotoxic therapy. Similarly, genes can be designed and introduced to modulate key processes in glioma growth and the body's response to it, such as angiogenesis and the host immune response—both of which play a key role in disease progression.^11^, ^12^

However, as is the case in many areas of biomedical research, the promising results from preclinical animal models have failed to be translated effectively into the clinic.^13^, ^14^, ^15^ This is exemplified by two unsuccessful phase III randomized controlled trials of gene therapy^16^, ^17^ and variable responses in other smaller clinical studies.^6^, ^18^, ^19^, ^20^, ^21^

Several narrative reviews have described the promise of gene therapy in malignant glioma^7^, ^22^, ^23^, ^24^; however, thus far this promise has remained unfulfilled in the clinical setting. Three complementary reasons for this failure have been proposed: (1) efficacy is overstated in animal models, (2) potential efficacy is understated in human clinical trials or (3) animal models simply do not recapitulate the human disease with sufficient fidelity in order to be useful. Systematic review and meta‐analysis can provide a more transparent and objective summary of a field of research than narrative reviews; they allow assessment of scientific rigour of included studies using standard instruments. In addition, stratified meta‐analysis can explore the impact of independent study design variables (termed external validity) on reported outcome; assess the prevalence and impact of measures to reduce bias such as randomization, blinded outcome assessment and sample size calculations (termed internal validity); and can provide evidence of possible publication bias—a phenomenon where comparative over‐reporting of small efficacious studies versus small ineffective studies leads to a false overestimation of the benefit of a given therapy.^25^, ^26^ Several previous studies on experimental models of neurological disease have demonstrated the flaws in the internal validity of studies and have shown that reporting of such measures can significantly affect efficacy estimates.^15^, ^27^, ^28^, ^29^ Consequently, assessing these features forms a critical domain of the systematic review and meta‐analysis in preclinical literature.

We hypothesized that limitations in the internal and external validity in animal modelling lead to an overstatement of efficacy of gene therapy in animal models of glioma. Here we use systematic review and meta‐analysis to describe the relationship between study design, study quality and the reported improvements in median survival, and the fidelity with which limitations in the animal data were taken into account in the design of human clinical trials.

## Methods

### literature search and inclusion criteria

Relevant full publications and meeting abstracts were identified by electronic searching of three online databases (Pubmed, Embase and Web of Knowledge) using the search terms: <gene therapy> AND <<glioma> OR <brain tumour> OR <brain tumour> OR <brain neoplasm> OR <glioblastoma> OR <ependymoma> OR <astrocytoma> OR <oligodendroglioma>>. Results were limited to animal studies with no language or date limits. Following comments raised in the review, we tested whether the term <gene therapy> was overly restrictive by searching for <glioma> and <thymidine kinase> to determine whether additional studies would be identified.

The inclusion criteria were adapted from previously published criteria^30^ and required studies to report: (1) a single form of gene therapy, (2) a rat or mouse model of glioma, (3) the glioma cell line used, (4) intracerebral implantation of the tumour, (5) median survival data reported within the text or which could be calculated from Kaplan Meier survival graphs and (6) the number of animals in the control and treatment group(s). We defined a single gene therapy as the use of a single vector containing either one or multiple genes. To improve the sensitivity of identification of relevant studies, each publication identified in the electronic search was assessed individually against the inclusion and exclusion criteria by two of four independent reviewers (SC, ALM, TCH and MRM), with differences resolved by discussion.

### methodological quality

A 9‐item quality checklist was adapted from the CAMARADES (Collaborative Approach to Meta‐Analysis and Review of Animal Data in Experimental Studies) published criteria^31^ and the glioma‐specific score previously described by our group.^30^ The checklist comprised (1) publication in a peer‐reviewed journal and the reporting of (2) the number of tumour cells implanted, (3) randomized allocation of tumour‐bearing animals to treatment and control groups, (4) blinded assessment of outcome, (5) a sample size calculation, (6) compliance with animal welfare regulations, (7) a potential conflict of interest, (8) the number of animals originally inoculated with tumour cells and (9) an explanation of any treated animals excluded from survival analysis. While not detailed as a quality checklist item in the study protocol, in response to comments raised in review we have also considered whether the study provided evidence of successful transduction and gene expression *in vitro*, and whether the study provided evidence of infection, replication and expression in the tumour *in vivo*.

### data extraction and analysis

We extracted data for median survival time, the number of animals in both the treatment and control groups and details of study design characteristics (the gene therapy used, animal species, co‐morbidities, tumour cell line, gene therapy vector, route of administration, number of doses, delay to treatment and method of determining survival and presentation of data (i.e. textual or graphical)). We grouped gene therapy into broad categories of angiogenesis, DNA repair, immunomodulation, oncolytic and “other”. We calculated an effect size for each comparison by dividing the median survival in the treatment group by the median survival in the control group to give a median survival ratio.

A preliminary stratification identified that studies that used a prodrug in the treatment group (to activate the gene therapy) but not in the control group were associated with significantly larger effects than those using prodrug in both groups, suggesting biological activity of the prodrug alone. Therefore, studies using a prodrug were only included where the same prodrug was also used in the control group. Some studies reported more than one control group. We considered the most appropriate control group to be the one that was most similar to the treatment group, while offering no functional gene therapy, according to a hierarchy that prodrug with non‐functioning vector was preferred to prodrug with saline, which was preferred to prodrug only. Where a prodrug was not used, the hierarchy was non‐functioning vector in preference to saline‐only in preference to no treatment. For studies in which more than 50% of animals survived till the end of the experiment, we used the last time point at which survival was reported to give a conservative measure of median survival.

Individual study effect sizes were weighted by the number of animals for that comparison, as there is no inherent measure of variance available for median survival data. Where a control group served more than one treatment group we corrected the weighting of the study by dividing the number of animals in the control group by the number of treatment groups served. Effect sizes were calculated on log‐transformed data^32^ using the random effects model of Dersimonian and Laird,^33^ as we expected significant heterogeneity between experiments. We estimated the standard error of the summary estimates from the inter‐study variance (as described previously^27^, ^34^).

We used stratified meta‐analysis to estimate the significance of differences between groups of studies by partitioning heterogeneity and using the χ^2^ distribution with n − 1 degrees of freedom (where n is the number of strata). We performed stratified analyses on the complete dataset that included all gene therapies reported. We also performed analyses on a more homogenous subset of data that consisted of only the most common gene therapy—herpes simplex virus thymidine kinase activated by ganciclovir (GCV). To allow for multiple comparisons (we performed 26 comparisons; 15 on the complete dataset and 11 on the thymidine kinase subset of data) we adjusted our significance level to p < 0.0019 using Bonferroni correction for 26 tests of statistical significance in the same dataset. We used funnel plotting,^35^ Egger regression^36^ and “trim and fill”^37^ to assess for the presence of publication bias.

To estimate the statistical power of a typical experiment, we calculated the median observed values for median survival in the control and treatment groups and the median numbers in each group and used the “stpower exponential” functional in Stata. Data extracted from studies included in the review and the results of meta‐analysis and publication bias assessment are available from the Dryad Digital Repository: http://doi.org/10.5061/dryad.bs8c4.^38^

## Results

**Figure 1:**
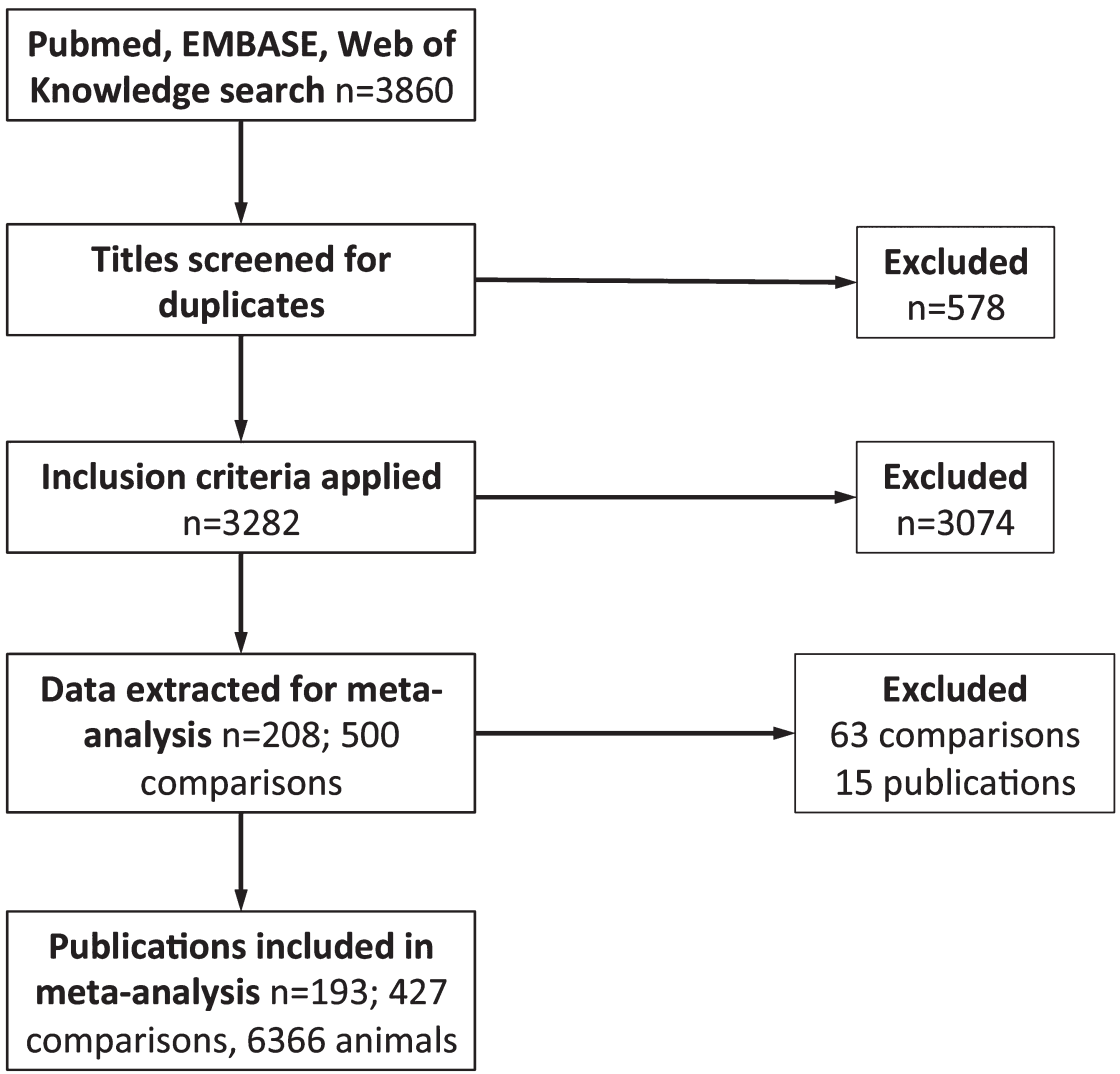
Study selection summary.

We identified 3,860 publications, of which 208 met our inclusion criteria (Figure 1; Appendix S1, Supporting Information). Of these, 193 publications reported data suitable for meta‐analysis; these described 427 comparisons using 6,366 animals (Appendices S1 and S2).

**Figure 2:**
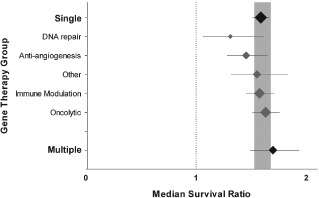
Stratification by gene therapy group. Grouping gene therapies into those using a single gene or those using multiple genes in a single vector did not account for between‐study heterogeneity (p > 0.0019, n = 387 and 40 respectively; black plots with bold labels). Furthermore, subcategorizing the single gene group by the broad mechanism of action of that gene did not account for between‐study heterogeneity (p < 0.0019, grey plots). Plots represent mean ± 95% confidence interval (CI) and the diamond represents a measure of number of comparisons within each stratum. The dotted line represents the level of neutral treatment effect.

Overall, 127 different gene therapies were tested. A total of 101 used a single gene and 26 used two genes in a single vector (Appendices S2 and S3). Thymidine kinase was the most common gene therapy (61 comparisons, given as a single gene therapy in 49) followed by IL‐4 (23 comparisons), IL‐2 (21 comparisons) and tumour necrosis factor‐related apoptosis inducing ligand (TRAIL, 18 comparisons). Across all 127 gene therapies there was a significant improvement in the median survival time (survival ratio 1.60, 95% CI 1.53–1.67), and there was significant between‐study heterogeneity (χ^2^ = 1,522; df = 426, p < 0.0019; I^2^ = 72%). While the approaches to gene therapy were diverse, the broad category of gene therapy used did not account for a significant proportion of the observed heterogeneity (Figure 2).

### risk of bias

The median number of quality checklist items reported was three of a possible nine (interquartile range (IQR) 3–4; Appendix S4); 193 (100%) publications were in peer‐reviewed journals, 170 (88.1%) reported the number of tumour cells implanted, 23 (12.4%) randomly allocated animals to group, 7 (3.6%) blinded the assessment of outcome, 133 (68.9%) had a statement of compliance with animal welfare regulations, 15 (7.8%) had a statement of a potential conflict of interest, 24 (12.4%) reported the number of animals originally inoculated with the tumour and 41 (21.1%) gave an explanation of any treated animals excluded from the survival analysis. A total of 139 studies (72.0%) provided evidence of successful transduction and gene expression *in vitro* and 90 (46.6%) provided evidence of infection, replication or expression in the tumour *in vivo*. No publication reported a sample size calculation and the median number of animals in each of the control and treatment groups was eight (IQR 6–10). In 90 publications it was reported that animals were killed when they manifested signs reflecting disease of a certain severity (rather than allowing them to die of their disease), and in the remainder of studies the circumstances of death (euthanasia or spontaneous) were not reported.

**Figure 3:**
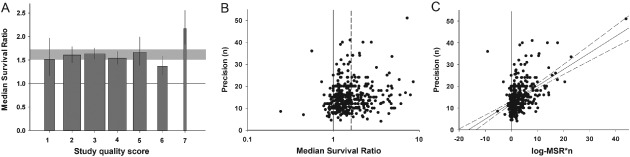
External validity. **A**. Stratification by aggregated quality score did not account for between‐study heterogeneity, implying no variation in efficacy with quality score in this dataset (p > 0.0019). The grey band represents global 95% confidence intervals (CIs); columns represent mean ± 95% CI and column width a measure of number of comparisons within each stratum. The solid line represents the level of neutral treatment effect. **B**. Funnel plots showing effect size (x‐axis) versus a measure of study precision (y‐axis). The dataset appears to be skewed, with imprecise studies generally showing more efficacy than those with larger sample sizes. The solid line represents the line of neutral treatment effect and the dotted line marks the global efficacy estimate. **C**. Egger regression plot depicting effect size × precision (x‐axis) versus precision (y‐axis). Regression revealed a positive intercept (p < 0.001) implying an excess of small, imprecise studies. The vertical solid line represents the level of neutral treatment effect; dotted lines represent 95% CI of the regression.

The aggregate number of quality checklist items scored or the reporting of randomized group allocation did not account for between‐study heterogeneity (Figure 3A). Only seven publications (9/427 comparisons) reported the blinded assessment of outcome, too few to allow further analysis. We did not identify any differences in treatment effects between studies that reported survival data within the text and those where data were extracted from a graph.

Bias introduced by an excess of small, imprecise studies was suggested with asymmetry in the funnel plot (Figure 3B) and Egger regression (11.3 ± 0.301; t = 11.3, p < 0.001; Figure 3C) but not using “trim and fill”.

### influence of factors relating to gene delivery

**Figure 4:**
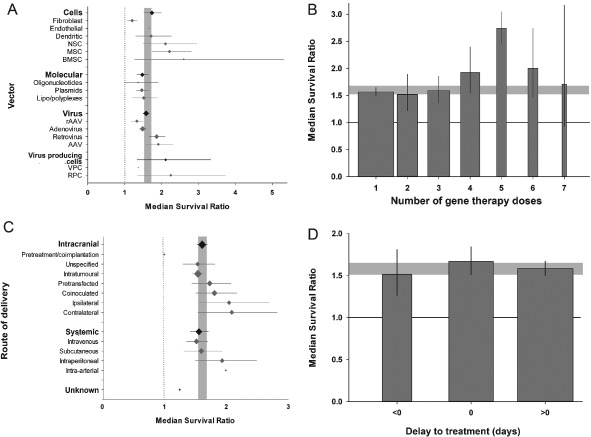
Features of gene therapy delivery. **A**. The vector used to deliver gene therapy accounted for a significant proportion of heterogeneity in the complete dataset, and there was evidence of differing efficacy between different cellular and virus vector paradigms (p < 0.0019). The grey band represents global 95% confidence intervals (CIs); plots represent mean ± 95% CI and the diamond represents a measure of number of comparisons within each stratum. The dotted line represents the level of neutral treatment effect. **B**. The number of doses accounted for between‐study heterogeneity (p < 0.0019), the largest effect seen with five doses. The grey band represents global 95% CIs; columns represent mean ± 95% CI and column width a measure of number of comparisons within each stratum. The solid line represents the level of neutral treatment effect. **C** The route of gene therapy delivery accounted for heterogeneity, with the largest efficacy associated with intra‐arterial and intraperitoneal systemic delivery, and ipsilateral and contralateral intracranial therapy (p < 0.0019). There was no observed difference in efficacy between intracranial and systemic administration. The grey band represents global 95% CIs; plots represent mean ± 95% CI and the diamond represents a measure of number of comparisons within each stratum. The dotted line represents the level of neutral treatment effect. **D**. The delay to treatment (where “0” refers to the day of tumour inoculation, “>0” therapy initiation post‐tumour inoculation and “<0” therapy initiated before tumour inoculation) accounted for heterogeneity (p < 0.0019), with therapy given concomitantly with tumour inoculation giving the greatest efficacy. The grey band represents global 95% CIs; columns represent mean ± 95% CI and column width a measure of number of comparisons within each stratum. The solid line represents the level of neutral treatment effect.

The method of gene delivery (molecules, viruses, cells or virus‐producing cells) had a significant impact on the reported effect size. Cells and virus‐producing cells were associated with the largest treatment effects (χ^2^ = 24.1, df = 3, p < 0.0019; Figure 4A). Furthermore, the selection of viral delivery system accounted for a significant proportion of between‐study heterogeneity (χ^2^ = 53.0, df = 3, p < 0.0019). The greatest estimates of effect were observed where retroviruses and adeno‐associated viruses were used. Similarly, selection of cellular delivery system also accounted for significant between‐study heterogeneity (χ^2^ = 56.2, df = 5, p < 0.0019). Bone marrow‐derived stem cells and neural stem cells were associated with the largest effect sizes.

Several gene therapy paradigms were reported that usually included the concomitant use of a prodrug; 67 comparisons reported such combinations, for instance thymidine kinase with GCV, cytosine deaminase (CD) with 5‐fluorocystine (5‐FC). Furthermore, these same gene therapies were sometimes used without the appropriate prodrug (35 comparisons). Gene therapies using a prodrug were associated with an increased effect size if that prodrug was used (1.90; 95% CI 1.67–2.14, n = 67) when compared with the same gene therapies where no prodrug was used (1.27; 1.14–1.41, n = 35; χ^2^ = 45.6, df = 1, p < 0.0019) with median survival ratio being 50% higher (95% CI 35 –65%). A further 17 comparisons involved prodrugs not usually associated with the gene therapy used (i.e. prodrug used alongside that gene in fewer than 50% of comparisons; e.g. interferon with 5‐FC, tumour necrosis factor with GCV). The most commonly used prodrugs were GCV (40 comparisons) and 5‐FC (24 comparisons, see Appendix S2).

The number of gene therapy doses administered (ranging from 1–7 doses) also accounted for a significant proportion of the between‐study heterogeneity. We observed a direct relationship between the number of doses and effect size where up to five doses were given followed by a fall in efficacy where six or seven doses were administered (χ^2^ = 50.1, df = 6, p < 0.0019; Figure 4B). Intracranial gene therapy delivery was common (328/427 comparisons) and we stratified these into intratumoural, ipsilateral (gene therapy introduced into the same cerebral hemisphere as the tumour), contralateral (opposite hemisphere), coinoculation (tumour and vector inoculated together), pretransfection (glioma cells transfected before tumour inoculation) and unspecified intracerebral. While these groups contain information on both location and time of implantation, we used a single stratification as the variables display colinearity (i.e. coinoculated cells can only be implanted at the same time as the tumour, intratumoural injection requires an established tumour to be present). Of these routes, intratumoural was the most common (226/328), followed by coinoculation (36/328) and pretransfection (31/328). The remaining comparisons, except for one unknown, were systemic—the most commonly used routes being subcutaneous (49/98) and intravenous (32/98). There was no difference in observed effect size between treatments that were delivered centrally and those that had to cross the blood–brain barrier (delivered systemically); however, we did observe significant portion of heterogeneity accounted for by more specific stratification of route of delivery (χ^2^ = 45.0, df = 11, p < 0.0019; Figure 4C). The routes associated with greatest efficacy were ipsilateral and contralateral central delivery, which were more effective than intratumoural treatment.

Gene therapy was delivered from 1 month before to 1 month after tumour induction. We stratified the data into three groups (before, the same day as, or after the induction of tumour), and the timing of treatment had a significant impact on reported efficacy (χ^2^ = 13.0, df = 2, p < 0.0019; Figure 4D). Where tumour cells were treated *in vitro* prior to implantation these were classified as therapy starting on the same day as implantation. The type of control group used did not account for any between‐study heterogeneity.

### influence of factors relating to the animal model used

**Figure 5:**
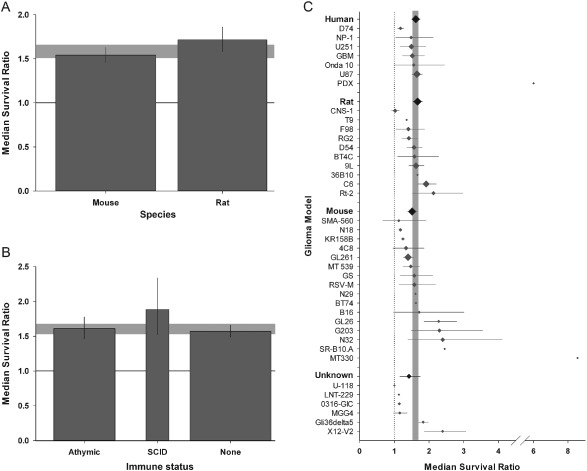
Glioma model setup. **A**. Gene therapy in rats was associated with greater efficacy than in mice (p < 0.0019). **B**. Immune status was associated with heterogeneity: use of animals with severe combined immunodeficiency were associated with greater efficacy than athymic or normal counterparts (p < 0.0019). The grey bands represent global 95% confidence intervals (CIs); columns represent mean ± 95% CI and column width a measure of number of comparisons within each stratum. The solid line represents the level of neutral treatment effect. **C**. Glioma model was associated with between‐study variance (p < 0.0019), with greatest efficacy seen with N32 and G203 lines in the complete dataset. Furthermore, the species of tumour origin was associated with heterogeneity (p < 0.0019). The grey band represents global 95% CIs; plots represent mean ± 95% CI and the diamond represents a measure of number of comparisons within each stratum. The dotted line represents the level of neutral treatment effect.

Overall, studies using rats reported significantly higher effect sizes (1.71; 95% CI 1.58–1.86; n = 144) than mice (1.54; 1.46–1.63; n = 283; χ^2^ = 13.0, df = 1, p < 0.0019; Figure 5A). Experiments were carried out in animals with no reported alterations of their immune status (310/427), athymic animals (92/427) and animals with various forms of severe combined immunodeficiency (SCID; 25/427); studies using SCID animals were associated with greater efficacy (χ^2^ = 16.2, df = 2, p < 0.0019; Figure 5B). Although the species of origin of the tumour line (mouse, rat, human or unknown) did not account for any between‐study heterogeneity, we observed an effect of the tumour line itself (χ^2^ = 244, df = 38, p < 0.0019; Figure 5C).

Median survival across all control groups was 25 days and across all treatment groups was 40 days. With a median of eight animals in control and treatment groups we estimate that the median powered study in this cohort has only 17% statistical power to detect the median change in median survival. This compares with around 30% in experimental stroke studies (CAMARADES group, data not published), and a convention of seeking power of 80 to 90% in well‐conducted clinical trials. As a guide for future investigators in Appendix S5, we present the relationship between statistical power and number needed per group for the above comparison and for a range of median survival ratios that might be sought for in future experiments.

### thymidine kinase dataset sub‐analysis

We performed a sensitivity analysis for the most common gene therapy paradigm (thymidine kinase with GCV, 30 comparisons using 446 animals). Thymidine kinase gene therapy was associated with a significant increase in median survival (1.99; 95% CI 1.68–2.37) and between‐study heterogeneity comparable with the complete dataset (χ^2^ = 49.9, df = 29, p < 0.0019; I^2^ = 69%).

**Figure 6:**
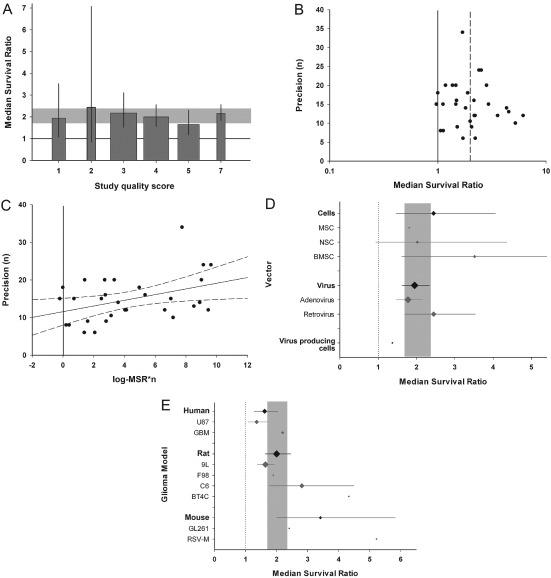
Thymidine kinase/HSV‐1 with ganciclovir (GCV) subset analysis. **A**. Stratification by aggregated quality score did not account for between‐study heterogeneity (p > 0.0019). The grey band represents global 95% confidence intervals (CIs); columns represent mean ± 95% CI and column width a measure of number of comparisons within each stratum. The solid line represents the level of neutral treatment effect. **B**. Funnel plots showing effect size (x‐axis) versus a measure of study precision (y‐axis). The dataset appears to be skewed, with imprecise studies generally showing more efficacy than those with larger sample sizes. The solid line represents the line of neutral treatment effect and the dotted line marks the global efficacy estimate. **C**. Egger regression plot depicting effect size*precision (x‐axis) versus precision (y‐axis). Regression revealed a positive intercept (p < 0.001). The vertical solid line represents the level of neutral treatment effect; dotted lines represent 95% CI of the regression. **D**. The vector used to deliver gene therapy was not associated with between‐study heterogeneity (p > 0.0019). **E**. The tumour line used accounted for heterogeneity (p < 0.0019), with RSV‐M, BT4C and C6 lines associated with greatest efficacy. However, we observed no difference in efficacy between cells originating from different species (p > 0.0019). The grey bands in D–E represent global 95% CIs; plots represent mean ± 95% CI and the diamond represents a measure of number of comparisons within each stratum. The dotted line represents the level of neutral treatment effect.

The risk of bias appears to be similar between the two datasets. The median study quality score was again 3 (IQR 2–3.25) and we did not find an association between the number of study quality checklist items scored and efficacy (Figure 6A). Small study bias was suggested with asymmetry in the funnel plot and a positive intercept on Egger regression (11.56 ± 1.76; t = 6.61, p < 0.001; Figure 6B and C), but not in “trim and fill” analysis. Again, there were no differences between studies that reported survival data within the text and those where data had to be extracted from a graph, or between the types of control used.

Only one study used more than one dose of gene therapy, only three used a route of delivery other than intracranial inoculation and only three reported efficacy in animals with co‐morbidity; these data were not analysed further. There was no effect of the method of gene delivery, the vector used (Figure 6D), the time to delivery, the species used or the species of origin of the tumour cell line.

The tumour line itself did however account for some of the observed heterogeneity in this dataset (χ^2^ = 40.4, df = 7, p < 0.0019; Figure 5E), as did the approach to determining survival (euthanasia, spontaneous death, unreported) (χ^2^ = 24.4, df = 2, p < 0.0019), with largest effect observed in studies reporting spontaneous death (median survival ratio 8%) (95% CI 1–15%) higher compared with studies killing animals when they became symptomatic).

Searching for <glioma> and <thymidine kinase> identified only one additional study. In a sensitivity analysis including data from this study, there were no changes of substance either in the point estimates of efficacy or in any of the conclusions drawn.

## Discussion

In this first systematic review and meta‐analysis of gene therapy in experimental glioma we show substantial and significant prolongation of median survival across a range of experimental conditions. However, there is a high risk of bias in included studies and we observed substantial heterogeneity; consequently these results should be interpreted with caution. We observed influences on reported efficacy by vector type, control type, delay to treatment, glioma model selection, animal, immune status and the method of determining survival. Further high‐quality preclinical investigation developed to better define the impact of the study design characteristics listed above, and in particular for those in which we identified substantial efficacy in conditions that reflect those seen in human disease, may provide promising avenues for clinical trial development.

### study quality

Overall the quality of studies was limited; the median number of quality checklist items scored was three of a possible nine (IQR 3–4). Our threshold for significance testing was conservative (Bonferroni correction for 26 stratifications), and while testing for an effect of study quality on treatment efficacy did not reach this significance threshold (p = 0.024), an association is likely. Only 12% of studies reported randomization and less than 4% reported the blinded assessment of outcome. As two thirds of studies met either three or four checklist items and none met more than seven, we have not been able to ascertain whether high‐quality studies give lower estimates of efficacy. This contrasts with findings from other models of neurological disease, where the prevalence of reporting of such factors is higher and where there is evidence that high‐quality studies report lower estimates of efficacy.^15^, ^39^, ^40^ We found some evidence of publication bias, including a positive intercept using Egger regression, but the trim and fill approach did not impute any theoretical missing studies. It has been suggested that trim and fill is less powerful than Egger regression,^36^ but the small study effects detected by Egger regression may have other causes, particularly where there is substantial heterogeneity between studies, as is the case here.^41^ Our findings are consistent with the presence of publication bias of the same order as reported in a previous systematic review of temozolomide in experimental glioma.^27^

### study design features affecting external validity

We found no differences between broad categories of gene therapies so we analysed all therapies together, with a sensitivity analysis using only the most commonly used therapy. It is likely that certain individual therapies were substantially more or less effective than the overall estimate but 79% of these were tested in fewer than four experiments (Appendix S3), and in these circumstances meta‐analysis contributes little that cannot be gleaned from an examination of primary data. Nonetheless, thymidine kinase was more efficacious than average (median survival ratio 1.99 vs. 1.60). While this supports there being differences in efficacy between treatments there are alternative explanations—for instance differences in the animal and glioma models used. We found differences in efficacy associated with different vector‐delivery mechanisms, routes of gene therapy delivery, numbers of doses and delays to treatment. Delivery of gene therapy using stem cells was associated with greatest efficacy in both datasets, and this might relate to more effective delivery to the required site of action, more sustained gene expression or other factors. In the complete dataset, intracranial delivery (rather than intralesional) and multiple dosing (particularly four or more doses) were most effective. In contrast to the difficulties of transfecting tumours in humans with glioma, we were surprised that, taken together, systemic therapies were as efficacious as those delivered intracranially.^42^, ^43^ Over 90% of the thymidine kinase studies administered gene therapy intracranially with a single dose—analogous to clinical practice.^16^, ^44^, ^45^, ^46^

We also found that features of the disease models used were widely variable and significantly affected the observed efficacy. A total of 40 different glioma cell lines were used, originating from humans, mice and rats. No studies used mice with spontaneously occurring or induced glioma cells and only one reported the use of cells recently extracted from human glioma specimens. Median survival ratios for different cell lines ranged from 1 to 8.56 (median 1.57; IQR 1.34–1.71). This suggests that cell line selection is one of the most important factors for investigators to consider during experimental design. As prominent were the species used and their immune status. Rats and mice were used in both datasets, and roughly one third of the animals used were immune‐suppressed; this was associated with greater efficacy. The immune system plays an important role in the body's response to glioma, so the immune‐compromised mouse may not be an ideal model of human disease.

Consistent with the modelling of other neurological conditions, these data suggest that the efficacy of gene therapies in glioma is characterized by heterogeneity in both the disease model used and the treatment delivery. Further, we observed a low prevalence of reporting of measures to reduce the risk of bias (see Appendix S4 for details), possible overstatement of efficacy in studies at risk of bias, and publication bias.^15^, ^29^, ^40^, ^47^, ^48^, ^49^

### potential weaknesses of our approach

There are a number of potential weaknesses to this approach. Foremost, meta‐analysis is essentially an observational technique. When we stratify by various study design characteristics and measures of study quality or bias, it may be that there are other unknown differences that are the cause of observed differences. For this reason we have been rigorous in only investigating sources of heterogeneity that we prespecified in a protocol. Our findings demonstrate association rather than causality; the observation that treatments delivered using cellular vectors are more effective than those using viruses or molecular approaches may be due to differences in gene delivery efficiency between these routes, or alternatively it may be that different types of interventions are more suited to different vector systems, and that these interventions differ as a class in their efficacy, or a difference in other study design characteristics shown to be associated with heterogeneity. For this reason our findings should be considered hypothesis‐generating only. However, further high‐quality preclinical studies can be used directly to test any hypothesis of interest.

Secondly, summarizing a field of research (as we have attempted here) requires by necessity the combination of data from experiments that are, to a greater or lesser extent, dissimilar. In these circumstances, the meaning of a summary estimate of efficacy across a range of studies has limited relevance other than to provide a yardstick of the magnitude of effect that might be expected of an intervention. However, we believe the statistical explanation of the differences between studies, especially in the face of the substantial heterogeneity observed here, is valid and important. This rationale is the basis on which we have deemed it appropriate, corroborated by the evidence that broad groups do not differ in efficacy, to collate all gene therapies into a single analysis—following this, heterogeneity was accounted for by measures of study design rather than the gene therapy paradigm itself. In support of these findings, we ran a separate analysis on the most commonly used gene therapy paradigm, thymidine kinase with GCV; in general we found that the same factors relating to study design and internal and external validity of these experiments had a significant impact on efficacy. Given the presence of such diversity in study design, our findings on study quality, randomization, controlling, experimental design and the consistency of these between the two datasets provide validation of our approach.

Our findings are only as reliable as the data on which they are based, and we have shown that this is likely to be confounded by poor study quality and by publication bias. However, our search strategy was broad, accepting conference abstracts and publications in languages other than English, so our approach is likely to provide a better summation of what is known than narrative reviews—which are subject to the same potential biases—and the impact of selection bias is likely to have reduced to the minimum possible. We used the term “gene therapy” in our search rather than detailing specific genes so that we might identify the largest number of studies, not just those where the use of that gene was already widely known. The term “gene therapy” may have been unduly restrictive, but searching for “thymidine kinase” and “glioma” in Pubmed—without further limitations—identified only one additional study, inclusion of data from which had no impact on the overall efficacy estimate of efficacy. Finally, we have attempted to minimize false positives in our statistical tests by adjusting for multiple comparisons.

The use of meta‐analysis to summarize median survival data is not well established. Because we did not have access to data for individual animals we could not pool hazard ratios as has been suggested for clinical studies,^50^ and instead have used methods reported previously,^27^, ^34^ based on the work of Simes et al.,^32^ as a summary estimate that is comparable with hazard ratio pooling.

### within these limitations, are there any implications for future research or for the design of clinical trials?

In this systematic review and meta‐analysis, we have presented substantial evidence that features relating to the risk of bias and experimental design of animal studies significantly affect the observed efficacy of gene therapy for experimental glioma. However, another issue yet to be addressed is that of construct validity; there is evidence from these data that translation of gene therapy from experimental to clinical glioma has failed because the experimental models do not recapitulate human disease.

The optimized conditions that are generally created for animal studies do not recapitulate the heterogeneity of human glioma patients, as these studies are all undertaken on homogeneous rodent populations. While techniques such as meta‐analysis seek to counteract this homogeneity, the breadth achieved still does not reflect that of the human population. For example, of these animals, many are immune‐compromised, a feature that is uncommon in clinical practice. We have observed a wide variety of glioma models used, but those most commonly selected (GL261, U87 and C6) tend to grow quickly and relatively non‐invasively into large discreet spheres,^51^, ^52^ contrasting sharply with irregularly shaped, poorly defined, infiltrative human glioblastoma multiforme tumours.^1^, ^8^ While each cell line has certain properties that do relate to human disease—for example extensive capillary networks in U87 models,^53^ white matter invasion and low immunogenicity in GL261,^54^, ^55^, ^56^ gene mutations in C6 that are comparable to human glioma^57^—these tumours are appropriate for the study of particular components of glioma biology (such as angiogenesis in U87 or immune therapies in GL261) but perhaps lack the robustness for survival studies preceding translation into clinical trial. The recent emergence of the glioma stem cell hypothesis (implicating a cell with stem‐like features in the aetiology and pathogenesis of human glioma) has influenced the design of novel preclinical models,^58^ but these, to our knowledge, have not yet been adopted into animal studies of gene therapy. Another novel practice is the use of patient‐derived xenografts, where animals are inoculated with tumour cells prepared from fresh human surgical specimens rather than cells from established *in vitro* cultures. These models may be more characteristic of human disease and provide genetic heterogeneity not seen with traditional glioma models;^59^ however, we identified only one relevant study using this approach. Finally in animal studies gene transfection rates are evidently high enough to be therapeutic, even when vectors are delivered systemically. Indeed, GL261 tumours are transfected very efficiently by adenoviruses.^60^ This contrasts with human therapy where transfection rates are low; the blood–brain barrier is obstructive in glioma therapeutics, preventing the use of systemic vector delivery,^43^, ^61^ even when vectors are delivered distal to the ophthalmic artery (Dr Robin Grant, personal communication), as tumour penetration is poor and side effects high. When implanted locally, distribution throughout the tumour is difficult to achieve.^43^ This may be attributable to differences in the central nervous system anatomy and the host immune system.^43^

The large between‐study heterogeneity observed in our data suggests that the efficacy of gene therapy is very variable, depending at least in part on the features we have described and perhaps to a greater degree than is observed in other glioma treatments.^27^, ^30^ This matches the so‐called lack of “robustness” seen with gene therapy in phase II and III clinical trials^42^ that has ultimately led to failure.

The statistical power of the experiments included in this meta‐analysis was low, and no study reported a formal sample size calculation. Improving the statistical power of gene therapy experiments may help to reduce heterogeneity by reducing the chances of type II (false negative) errors and also the predictive value of positive studies where the prior probability of success was low.^62^, ^63^ We hope the community finds our guide to statistical power (Appendix S5) helpful in the design of future experiments.

In spite of these limitations, gene therapy treatment for experimental glioma appears to be effective when initiated at later time points, and efficacy was observed against cells of human origin. Both these features are pertinent to successful treatment for human disease, as tumours are only discovered after a period of growth. Gene therapy was effective when given either intracranially or systemically although this does not seem to correlate with clinical experience. Efficacy appeared to be highest when five doses of the gene therapy were given, but—given the difficulties of systemic administration in humans—it may not be practicable to implant locally more than once. Exploration of the most effective number of treatments was not addressed in any of the included publications and is an important topic for further animal study.

As such, we recommend that future preclinical research focuses on genes ratified in both animal and human glioma cell biology, using orthotopic tumours and intracranial gene delivery over one or more doses; they should ideally use stem‐like cancer cells or patient‐derived xenografts, or at least provide a rationale for tumour model selection, in non‐immune‐compromised animals where possible. These studies should be registered, randomized, blind assessment of outcome and provide a sample size calculation in all but hypothesis‐generating experiments in accordance with ARRIVE guidelines.^64^

## Conclusions

Gene therapies are associated with substantial increases in median survival in animal models of cerebral glioma, but because of concerns about the internal (study quality), external (study design) and particularly construct (recapitulation of human disease) validity of this literature, these findings should be interpreted with caution. Our analysis suggests that a strategy based on multiple treatments with viral or cellular vectors expressing genes of interest delivered locally, tested in the potentially more relevant tumour models described recently, represents a plausible approach to developing gene therapies for glioma. However, the issues of study quality and construct validity of existing models that we have identified here should be addressed in further animal studies if such strategies are to have the best chance of success.

## Conflict of Interest

The authors disclose no potential conflicts of interest for this article.

## Supporting information

Appendix S1. References used in systematic review.

Appendix S2. Study characteristics.

Appendix S3. Collated gene therapies.

Appendix S4. Study quality scores.

Appendix S5. Number of animals per group needed to achieve a set statistical power.

## References

[1] Anderson E , Grant R , Lewis SC , Whittle IR . Randomized phase III controlled trials of therapy in malignant glioma: where are we after 40 years? Br. J. Neurosurg. 2008; 22: 339–349. 18568722 10.1080/02688690701885603

[2] Stupp R , Mason WP , Van Den Bent MJ *et al.* Radiotherapy plus concomitant and adjuvant temozolomide for glioblastoma. N. Engl. J. Med. 2005; 352: 987–996. 15758009 10.1056/NEJMoa043330

[3] Andersen AP . Postoperative irradiation of glioblastomas. Results in a randomized series. Acta Radiol. Oncol. Radiat. Phys. Biol. 1978; 17: 475–484. 216238 10.3109/02841867809128178

[4] Walker MD , Strike TA , Sheline GE . An analysis of dose‐effect relationship in the radiotherapy of malignant gliomas. Int. J. Radiat. Oncol. Biol. Phys. 1979; 5: 1725–1731. 231022 10.1016/0360-3016(79)90553-4

[5] Stewart LA . Chemotherapy in adult high‐grade glioma: a systematic review and meta‐analysis of individual patient data from 12 randomised trials. Lancet 2002; 359: 1011–1018. 11937180 10.1016/s0140-6736(02)08091-1

[6] Natsume A , Yoshida J . Gene therapy for high‐grade glioma: current approaches and future directions. Cell Adh. Migr. 2008; 2: 186–191. 19262115 10.4161/cam.2.3.6278 PMC2634089

[7] Sasaki M , Plate KH . Gene therapy of malignant glioma: recent advances in experimental and clinical studies. Ann. Oncol. 1998; 9: 1155–1166. 9862044 10.1023/a:1008488709359

[8] Jellinger K . Glioblastoma multiforme: morphology and biology. Acta Neurochir. (Wien) 1978; 42: 5–32. 211808 10.1007/BF01406628

[9] Martuza RL , Malick A , Markert JM , Ruffner KL , Coen DM . Experimental therapy of human glioma by means of a genetically engineered virus mutant. Science 1991; 252: 854–856. 1851332 10.1126/science.1851332

[10] Markert JM , Coen DM , Malick A , Mineta T , Martuza RL . Expanded spectrum of viral therapy in the treatment of nervous system tumors. J. Neurosurg. 1992; 77: 590–594. 1326612 10.3171/jns.1992.77.4.0590

[11] Parney IF . Basic concepts in glioma immunology. Adv. Exp. Med. Biol. 2012; 746: 42–52. 22639158 10.1007/978-1-4614-3146-6_4

[12] Chi AS , Sorensen AG , Jain RK , Batchelor TT . Angiogenesis as a therapeutic target in malignant gliomas. Oncologist 2009; 14: 621–636. 19487335 10.1634/theoncologist.2008-0272 PMC4790121

[13] O'Collins VE , Donnan GA , Macleod MR , Howells DW . Scope of preclinical testing versus quality control within experiments. Stroke 2009; 40: e497. 19498189 10.1161/STROKEAHA.109.550335

[14] Van Der Worp HB , Howells DW , Sena ES *et al.* Can animal models of disease reliably inform human studies? PLoS Med. 2010; 7: e1000245. 20361020 10.1371/journal.pmed.1000245 PMC2846855

[15] Vesterinen HM , Sena ES , French‐Constant C , Williams A , Chandran S , Macleod MR . Improving the translational hit of experimental treatments in multiple sclerosis. Mult. Scler. 2010; 16: 1044–1055. 20685763 10.1177/1352458510379612

[16] Rainov NG . A phase III clinical evaluation of herpes simplex virus type 1 thymidine kinase and ganciclovir gene therapy as an adjuvant to surgical resection and radiation in adults with previously untreated glioblastoma multiforme. Hum. Gene Ther. 2000; 11: 2389–2401. 11096443 10.1089/104303400750038499

[17] Westphal M , Yla‐Herttuala S , Martin J *et al.* Adenovirus‐mediated gene therapy with sitimagene ceradenovec followed by intravenous ganciclovir for patients with operable high‐grade glioma (ASPECT): a randomised, open‐label, phase 3 trial. Lancet Oncol. 2013; 14: 823–833. 23850491 10.1016/S1470-2045(13)70274-2

[18] Lang FF , Bruner JM , Fuller GN *et al.* Phase I trial of adenovirus‐mediated p53 gene therapy for recurrent glioma: biological and clinical results. J. Clin. Oncol. 2003; 21: 2508–2518. 12839017 10.1200/JCO.2003.21.13.2508

[19] Pulkkanen KJ , Yla‐Herttuala S . Gene therapy for malignant glioma: current clinical status. Mol. Ther. 2005; 12: 585–598. 16095972 10.1016/j.ymthe.2005.07.357

[20] Ram Z , Culver KW , Oshiro EM *et al.* Therapy of malignant brain tumors by intratumoral implantation of retroviral vector‐producing cells. Nat. Med. 1997; 3: 1354–1361. 9396605 10.1038/nm1297-1354

[21] Shand N , Weber F , Mariani L *et al.* A phase 1–2 clinical trial of gene therapy for recurrent glioblastoma multiforme by tumor transduction with the herpes simplex thymidine kinase gene followed by ganciclovir. GLI328 European‐Canadian Study Group. Hum. Gene Ther. 1999; 10: 2325–2335. 10515452 10.1089/10430349950016979

[22] Kaufmann JK , Chiocca EA . Glioma virus therapies between bench and bedside. Neuro Oncol. 2014; 16: 334–351. 24470549 10.1093/neuonc/not310 PMC3922526

[23] Murphy AM , Rabkin SD . Current status of gene therapy for brain tumors. Transl. Res. 2013; 161: 339–354. 23246627 10.1016/j.trsl.2012.11.003 PMC3733107

[24] Whittle IR . Gene therapy for brain tumours. Br. J. Neurosurg. 1995; 9: 717–720. 8719825 10.1080/02688699550040666

[25] Ioannidis JP , Lau J . Pooling research results: benefits and limitations of meta‐analysis. Jt. Comm. J. Qual. Improv. 1999; 25: 462–469. 10481815 10.1016/s1070-3241(16)30460-6

[26] Macleod MR , O'Collins T , Howells DW , Donnan GA . Pooling of animal experimental data reveals influence of study design and publication bias. Stroke 2004; 35: 1203–1208. 15060322 10.1161/01.STR.0000125719.25853.20

[27] Hirst TC , Vesterinen HM , Sena ES , Egan KJ , Macleod MR , Whittle IR . Systematic review and meta‐analysis of temozolomide in animal models of glioma: was clinical efficacy predicted? Br. J. Cancer 2013; 108: 64–71. 23321511 10.1038/bjc.2012.504 PMC3553514

[28] Sena ES , Briscoe CL , Howells DW , Donnan GA , Sandercock PA , Macleod MR . Factors affecting the apparent efficacy and safety of tissue plasminogen activator in thrombotic occlusion models of stroke: systematic review and meta‐analysis. J. Cereb. Blood Flow Metab. 2010a; 30: 1905–1913. 20648038 10.1038/jcbfm.2010.116 PMC3002882

[29] Sena ES , Van Der Worp HB , Bath PM , Howells DW , Macleod MR . Publication bias in reports of animal stroke studies leads to major overstatement of efficacy. PLoS Biol. 2010b; 8: e1000344. 20361022 10.1371/journal.pbio.1000344 PMC2846857

[30] Amarasingh S , Macleod MR , Whittle IR . What is the translational efficacy of chemotherapeutic drug research in neuro‐oncology? A systematic review and meta‐analysis of the efficacy of BCNU and CCNU in animal models of glioma. J. Neurooncol 2009; 91: 117–125. 18813876 10.1007/s11060-008-9697-z

[31] Sena E , Van Der Worp HB , Howells D , Macleod M . How can we improve the pre‐clinical development of drugs for stroke? Trends Neurosci. 2007a; 30: 433–439. 17765332 10.1016/j.tins.2007.06.009

[32] Simes RJ . Confronting publication bias: a cohort design for meta‐analysis. Stat. Med. 1987; 6: 11–29. 3576013 10.1002/sim.4780060104

[33] Dersimonian R , Laird N . Meta‐analysis in clinical trials. Control. Clin. Trials 1986; 7: 177–188. 3802833 10.1016/0197-2456(86)90046-2

[34] Vesterinen HM , Sena ES , Egan KJ *et al.* Meta‐analysis of data from animal studies: a practical guide. J. Neurosci. Methods 2014; 221: 92–102. 24099992 10.1016/j.jneumeth.2013.09.010

[35] Light RJ , Pillemer DB . Summing up: the science of reviewing research. Cambridge, MA: Harvard University Press, 1984.

[36] Egger M , Davey Smith G , Schneider M , Minder C . Bias in meta‐analysis detected by a simple, graphical test. BMJ 1997; 315: 629–634. 9310563 10.1136/bmj.315.7109.629 PMC2127453

[37] Duval S , Tweedie R . Trim and fill: a simple funnel‐plot‐based method of testing and adjusting for publication bias in meta‐analysis. Biometrics 2000; 56: 455–463. 10877304 10.1111/j.0006-341x.2000.00455.x

[38] Hirst TC , Vesterinen HM , Conlin S , Egan KJ , Antonic A , Lawson McLean A , Macleod MR , Grant R , Brennan PM , Sena ES , Whittle IR . A systematic review and meta-analysis of gene therapy in animal models of cerebral glioma: why did promise not translate to human therapy? Dryad Digital Repository. Available at: http://doi.org/10.5061/dryad.bs8c4. 10.1002/ebm2.6 PMC5020579 27668084

[39] Frantzias J , Sena ES , Macleod MR , Al‐Shahi Salman R . Treatment of intracerebral hemorrhage in animal models: meta‐analysis. Ann. Neurol. 2011; 69: 389–399. 21387381 10.1002/ana.22243

[40] Van Der Worp HB , Sena ES , Donnan GA , Howells DW , Macleod MR . Hypothermia in animal models of acute ischaemic stroke: a systematic review and meta‐analysis. Brain 2007; 130: 3063–3074. 17478443 10.1093/brain/awm083

[41] Duval S , Weinhandl E . Correcting for publication bias in the presence of covariates. Rockville, MD: Agency for Healthcare Research and Quality, 2011. 22359778

[42] Lowenstein PR , Castro MG . Uncertainty in the translation of preclinical experiments to clinical trials. Why do most phase III clinical trials fail? Curr. Gene Ther. 2009; 9: 368–374. 19860651 10.2174/156652309789753392 PMC2864134

[43] Tobias A , Ahmed A , Moon KS , Lesniak MS . The art of gene therapy for glioma: a review of the challenging road to the bedside. J. Neurol. Neurosurg. Psychiatry 2013; 84: 213–222. 22993449 10.1136/jnnp-2012-302946 PMC3543505

[44] Assi H , Candolfi M , Baker G , Mineharu Y , Lowenstein PR , Castro MG . Gene therapy for brain tumors: basic developments and clinical implementation. Neurosci. Lett. 2012; 527: 71–77. 22906921 10.1016/j.neulet.2012.08.003 PMC3462660

[45] Sandmair AM , Loimas S , Puranen P *et al.* Thymidine kinase gene therapy for human malignant glioma, using replication‐deficient retroviruses or adenoviruses. Hum. Gene Ther. 2000; 11: 2197–2205. 11084677 10.1089/104303400750035726

[46] Smitt PS , Driesse m , Wolbers J , Kros M , Avezaat C . Treatment of relapsed malignant glioma with an adenoviral vector containing the herpes simplex thymidine kinase gene followed by ganciclovir. Mol. Ther. 2003; 7: 851–858. 12788659 10.1016/s1525-0016(03)00100-x

[47] Macleod MR , Van Der Worp HB , Sena ES , Howells DW , Dirnagl U , Donnan GA . Evidence for the efficacy of NXY‐059 in experimental focal cerebral ischaemia is confounded by study quality. Stroke 2008; 39: 2824–2829. 18635842 10.1161/STROKEAHA.108.515957

[48] Rooke ED , Vesterinen HM , Sena ES , Egan KJ , Macleod MR . Dopamine agonists in animal models of Parkinson's disease: a systematic review and meta‐analysis. Parkinsonism Relat. Disord. 2011; 17: 313–320. 21376651 10.1016/j.parkreldis.2011.02.010

[49] Sena E , Wheble P , Sandercock P , Macleod M . Systematic review and meta‐analysis of the efficacy of tirilazad in experimental stroke. Stroke 2007b; 38: 388–394. 17204689 10.1161/01.STR.0000254462.75851.22

[50] Michiels S , Piedbois P , Burdett S , Syz N , Stewart L , Pignon JP . Meta‐analysis when only the median survival times are known: a comparison with individual patient data results. Int. J. Technol. Assess. Health Care 2005; 21: 119–125. 15736523 10.1017/s0266462305050154

[51] Chen L , Zhang Y , Yang J , Hagan JP , Li M . Vertebrate animal models of glioma: understanding the mechanisms and developing new therapies. Biochim. Biophys. Acta 2013; 1836: 158–165. 23618720 10.1016/j.bbcan.2013.04.003 PMC3729034

[52] Kaye AH , Morstyn G , Gardner I , Pyke K . Development of a xenograft glioma model in mouse brain. Cancer Res. 1986; 46: 1367–1373. 3943101

[53] Strojnik T , Kavalar R , Lah TT . Experimental model and immunohistochemical analyses of U87 human glioblastoma cell xenografts in immunosuppressed rat brains. Anticancer Res 2006; 26: 2887–2900. 16886610

[54] Maes W , Van Gool SW . Experimental immunotherapy for malignant glioma: lessons from two decades of research in the GL261 model. Cancer Immunol. Immunother. 2011; 60: 153–160. 21120655 10.1007/s00262-010-0946-6 PMC11028904

[55] Leten C , Struys T , Dresselaers T , Himmelreich U . In vivo and ex vivo assessment of the blood brain barrier integrity in different glioblastoma animal models. J. Neurooncol 2014; 119: 297–306. 24990826 10.1007/s11060-014-1514-2

[56] Ausman JI , Shapiro WR , Rall DP . Studies on the chemotherapy of experimental brain tumors: development of an experimental model. Cancer Res. 1970; 30: 2394–2400. 5475483

[57] Barth RF , Kaur B . Rat brain tumor models in experimental neuro‐oncology: the C6, 9 L, T9, RG2, F98, BT4C, RT‐2 and CNS‐1 gliomas. J. Neurooncol 2009; 94: 299–312. 19381449 10.1007/s11060-009-9875-7 PMC2730996

[58] Singh SK , Hawkins C , Clarke ID *et al.* Identification of human brain tumour initiating cells. Nature 2004; 432: 396–401. 15549107 10.1038/nature03128

[59] Joo KM , Kim J , Jin J *et al.* Patient‐specific orthotopic glioblastoma xenograft models recapitulate the histopathology and biology of human glioblastomas in situ. Cell Rep. 2013; 3: 260–273. 23333277 10.1016/j.celrep.2012.12.013

[60] Szatmari T , Lumniczky K , Desaknai S *et al.* Detailed characterization of the mouse glioma 261 tumor model for experimental glioblastoma therapy. Cancer Sci. 2006; 97: 546–553. 16734735 10.1111/j.1349-7006.2006.00208.x PMC11159227

[61] Neuwelt EA . Reversible osmotic blood–brain barrier disruption in humans: implications for the chemotherapy of malignant brain tumors. Neurosurgery 1980; 7: 204. 6252507 10.1097/00006123-198008000-00018

[62] Ioannidis JP . Why most published research findings are false. PLoS Med. 2005; 2: e124. 16060722 10.1371/journal.pmed.0020124 PMC1182327

[63] Wacholder S , Chanock S , Garcia‐Closas M , El Ghormli L , Rothman N . Assessing the probability that a positive report is false: an approach for molecular epidemiology studies. J. Natl. Cancer Inst. 2004; 96: 434–442. 15026468 10.1093/jnci/djh075 PMC7713993

[64] Kilkenny C , Browne WJ , Cuthill IC , Emerson M , Altman DG . Improving bioscience research reporting: the ARRIVE guidelines for reporting animal research. PLoS Biol. 2010; 8: e1000412. 20613859 10.1371/journal.pbio.1000412 PMC2893951

