# Supplementary material for: A systematic review and meta‐analysis of gene therapy in animal models of cerebral glioma: why did promise not translate to human therapy?
Source: Evid Based Preclin Med. 2015 Jan 20;1(1):e00006. doi: 10.1002/ebm2.6 (PMC5020579; doi:10.1002/ebm2.6)
Supplement: Supplementary file 1 — Appendix S1. References used in systematic review. [file EBM2-1-21-s004.pdf]

Supplementary material 1: References used in systematic review

1. Adachi Y, Tamiya T, Ichikawa T, Terada K, Ono Y, Matsumoto K, et al. Experimental gene therapy for brain tumors using adenovirus-mediated transfer of cytosine deaminase gene and uracil phosphoribosyltransferase gene with 5-fluorocytosine. Human Gene Therapy11(1)()(pp 77-89), 2000Date of Publication: 2000. 2000:77-89.

2. Aghi M, Rabkin S, Martuza RL. Effect of chemotherapy-induced DNA repair on oncolytic herpes simplex viral replication. J NatlCancer Inst. 2006;98:38-50.

3. Ahmed AU, Thaci B, Alexiades NG, Han Y, Qian S, Liu F, et al. Neural stem cell-based cell carriers enhance therapeutic efficacy of an oncolytic adenovirus in an orthotopic mouse model of human glioblastoma. MolTher. 2011;19:1714-26.

4. Ahmed N, Salsman VS, Kew Y, Shaffer D, Powell S, Zhang YJ, et al. HER2-specific T cells target primary glioblastoma stem cells and induce regression of autologous experimental tumors. Clin Cancer Res. 2010;16:474-85.

5. Ali S, King GD, Curtin JF, Candolfi M, Xiong W, Liu C, et al. Combined immunostimulation and conditional cytotoxic gene therapy provide long-term survival in a large glioma model. Cancer Res. 2005;65:7194-204.

6. Allen C, Paraskevakou G, Iankov I, Giannini C, Schroeder M, Sarkaria J, et al. Interleukin-13 displaying retargeted oncolytic measles virus strains have significant activity against gliomas with improved specificity. MolTher. 2008;16:1556-64.

7. Alonso MM, Cascallo M, Gomez-Manzano C, Jiang H, Bekele BN, Perez-Gimenez A, et al. ICOVIR-5 shows E2F1 addiction and potent antiglioma effect in vivo. Cancer Res. 2007;67:8255-63.

8. Alonso MM, Jiang H, Yokoyama T, Xu J, Bekele NB, Lang FF, et al. Delta-24-RGD in combination with RAD001 induces enhanced anti-glioma effect via autophagic cell death. Molecular Therapy16(3)()(pp 487-493), 2008Date of Publication: Mar 2008. 2008:487-93.

9. Altanerova V, Cihova M, Babic M, Rychly B, Ondicova K, Mravec B, et al. Human adipose tissue-derived mesenchymal stem cells expressing yeast cytosinedeaminase::uracil phosphoribosyltransferase inhibit intracerebral rat glioblastoma. IntJCancer. 2012;130:2455-63.

10. Amano T, Kajiwara K, Yoshikawa K, Morioka J, Nomura S, Fujisawa H, et al. Antitumor effects of vaccination with dendritic cells transfected with modified receptor for hyaluronan-mediated motility mRNA in a mouse glioma model. J Neurosurg. 2007;106:638-45.

11. Ambar BB, Frei K, Malipiero U, Morelli AE, Castro MG, Lowenstein PR, et al. Treatment of experimental glioma by administration of adenoviral vectors expressing Fas ligand. Human Gene Therapy10(10)()(pp 1641-1648), 1999Date of Publication: 01 Jul 1999. 1999:1641-8.

12. Andreansky S, He B, Van CJ, McGhee J, Markert JM, Gillespie GY, et al. Treatment of

intracranial gliomas in immunocompetent mice using herpes simplex viruses that express murine interleukins. Gene Therapy5(1)()(pp 121-130), 1998Date of Publication: 1998. 1998:121-30.

13. Badie B, Kramar MH, Lau R, Boothman DA, Economou JS, Black KL. Adenovirus-mediated p53 gene delivery potentiates the radiation-induced growth inhibition of experimental brain tumors. Journal of Neuro-Oncology37(3)()(pp 217-222), 1998Date of Publication: 1998. 1998:217-22.

14. Benedetti S, DiMeco F, Pollo B, Cirenei N, Colombo BM, Bruzzone MG, et al. Limited efficacy of the HSV-TK/GCV system for gene therapy of malignant gliomas and perspectives for the combined transduction of the interleukin-4 gene. Human Gene Therapy8(11)()(pp 1345-1353), 1997Date of Publication: 20 Jul 1997. 1997:1345-53.

15. Benedetti S, Pirola B, Poliani PL, Cajola L, Pollo B, Bagnati R, et al. Dexamethasone inhibits the anti-tumor effect of interleukin 4 on rat experimental gliomas. Gene Therapy10(2)()(pp 188-192), 2003Date of Publication: Jan 2003. 2003:188-92.

16. Benedetti S, Pirola B, Pollo B, Magrassi L, Bruzzone MG, Rigamonti D, et al. Gene therapy of experimental brain tumors using neural progenitor cells. Nature Medicine6(4)()(pp 447-450), 2000Date of Publication: Apr 2000. 2000:447-50.

17. Berenstein M, Adris S, Ledda F, Wolfmann C, Medina J, Bravo A, et al. Different efficacy of in vivo herpes simplex virus thymidine kinase gene transduction and ganciclovir treatment on the inhibition of tumor growth of murine and human melanoma cells and rat glioblastoma cells. Cancer Gene Ther. 1999;6:358-66.

18. Bourbeau D, Lau CJ, Jaime J, Koty Z, Zehntner SP, Lavoie G, et al. Improvement of antitumor activity by gene amplification with a replicating but nondisseminating adenovirus. Cancer Res. 2007;67:3387-95.

19. Boviatsis EJ, Park JS, Sena-Esteves M, Kramm CM, Chase M, Efird JT, et al. Long-term survival of rats harboring brain neoplasms treated with ganciclovir and a herpes simplex virus vector that retains an intact thymidine kinase gene. Cancer Research54(22)()(pp 5745-5751), 1994Date of Publication: 15 Nov 1994. 1994:5745-51.

20. Boviatsis EJ, Scharf JM, Chase M, Harrington K, Kowall NW, Breakefield XO, et al. Antitumor activity and reporter gene transfer into rat brain neoplasms inoculated with herpes simplex virus vectors defective in thymidine kinase or ribonucleotide reductase. Gene Ther. 1994;1:323-31.

21. Bowers G, He J, Schulz K, Olivarria G, Maneval D, Olson JJ. Efficacy of adenoviral p53 delivery with SCH58500 in the intracranial 9l and RG2 models. Front Biosci. 2003;8:a54-a61.

22. Broaddus WC, Liu Y, Steele LL, Gillies GT, Lin PS, Loudon WG, et al. Enhanced radiosensitivity of malignant glioma cells after adenoviral p53 transduction. Journal of Neurosurgery91(6)()(pp 997-1004), 1999Date of Publication: Dec 1999. 1999:997-1004.

23. Candolfi M, Xiong W, Yagiz K, Liu C, Muhammad AK, Puntel M, et al. Gene therapy-mediated delivery of targeted cytotoxins for glioma therapeutics. ProcNatlAcadSciUSA. 2010;107:20021-6.

24. Candolfi M, Yagiz K, Foulad D, Alzadeh GE, Tesarfreund M, Muhammad AK, et al. Release of HMGB1 in response to proapoptotic glioma killing strategies: efficacy and neurotoxicity. Clin Cancer Res. 2009;15:4401-14.

25. Chambers R, Gillespie GY, Soroceanu L, Andreansky S, Chatterjee S, Chou J, et al. Comparison of genetically engineered herpes simplex viruses for the treatment of brain tumors in a scid mouse model of human malignant glioma. Proceedings of the National Academy of Sciences of the United States of America92(5)()(pp 1411-1415), 1995Date of Publication: 28 Feb 1995. 1995:1411-5.

26. Chen J, Wang ZR, Li H, Wei YQ, Wang W, Zhu B. An experimental research on the combination treatment of sFLK-1 gene therapy combined with gamma knife surgery. [Chinese]. Journal of Sichuan University (Medical Science Edition)37(5)()(pp 708-711+716), 2006Date of Publication: Sep 2006. 2006:708-11+16.

27. Choi SA, Hwang SK, Wang KC, Cho BK, Phi JH, Lee JY, et al. Therapeutic efficacy and safety of TRAIL-producing human adipose tissue-derived mesenchymal stem cells against experimental brainstem glioma. Neuro Oncol. 2011;13:61-9.

28. Ciesielski MJ, Apfel L, Barone TA, Castro CA, Weiss TC, Fenstermaker RA. Antitumor effects of a xenogeneic survivin bone marrow derived dendritic cell vaccine against murine GL261 gliomas. Cancer ImmunolImmunother. 2006;55:1491-503.

29. Cirielli C, Inyaku K, Capogrossi MC, Yuan X, Williams JA. Adenovirus-mediated wild-type p53 expression induces apoptosis and suppresses tumorigenesis of experimental intracranial human malignant glioma. J Neurooncol. 1999;43:99-108.

30. Conrad C, Miller CR, Ji Y, Gomez-Manzano C, Bharara S, McMurray JS, et al. Delta24-hyCD adenovirus suppresses glioma growth in vivo by combining oncolysis and chemosensitization. Cancer Gene Therapy12(3)()(pp 284-294), 2005Date of Publication: Mar 2005. 2005:284-94.

31. Cool V, Pirotte B, Gerard C, Dargent JL, Baudson N, Levivier M, et al. Curative potential of herpes simplex virus thymidine kinase gene transfer in rats with 9L gliosarcoma. HumGene Ther. 1996;7:627-35.

32. Curtin JF, Liu N, Candolfi M, Xiong W, Assi H, Yagiz K, et al. HMGB1 mediates endogenous TLR2 activation and brain tumor regression. PLoS Med. 2009;6:e10.

33. Denbo JW, Williams RF, Orr WS, Sims TL, Ng CY, Zhou J, et al. Continuous local delivery of interferon-beta stabilizes tumor vasculature in an orthotopic glioblastoma xenograft resection model. Surgery. 2011;150:497-504.

34. DiMeco F, Rhines LD, Hanes J, Tyler BM, Brat D, Torchiana E, et al. Paracrine delivery

of IL-12 against intracranial 9L gliosarcoma in rats. J Neurosurg. 2000;92:419-27.

35. Dmitrieva N, Yu L, Viapiano M, Cripe TP, Chiocca EA, Glorioso JC, et al. Chondroitinase ABC I-mediated enhancement of oncolytic virus spread and antitumor efficacy. ClinCancer Res. 2011;17:1362-72.

36. Dong Y, Wen P, Manome Y, Parr M, Hirshowitz A, Chen L, et al. In vivo replication-deficient adenovirus vector-mediated transduction of the cytosine deaminase gene sensitizes glioma cells to 5-fluorocytosine. HumGene Ther. 1996;7:713-20.

37. Ehtesham M, Samoto K, Kabos P, Acosta FL, Gutierrez MA, Black KL, et al. Treatment of intracranial glioma with in situ interferon-gamma and tumor necrosis factor-alpha gene transfer. Cancer Gene Ther. 2002;9:925-34.

38. Frankel B, Longo SL, Kyle M, Canute GW, Ryken TC. Tumor Fas (APO-1/CD95) up-regulation results in increased apoptosis and survival times for rats with intracranial malignant gliomas. Neurosurgery. 2001;49:168-75.

39. Friese MA, Platten M, Lutz SZ, Naumann U, Aulwurm S, Bischof F, et al. MICA/NKG2D-mediated immunogene therapy of experimental gliomas. Cancer Res. 2003;63:8996-9006.

40. Fueyo J, Alemany R, Gomez-Manzano C, Fuller GN, Khan A, Conrad CA, et al. Preclinical characterization of the antiglioma activity of a tropism-enhanced adenovirus targeted to the retinoblastoma pathway. J NatlCancer Inst. 2003;95:652-60.

41. Galipeau J, Li H, Paquin A, Sicilia F, Karpati G, Nalbantoglu J. Vesicular stomatitis virus G pseudotyped retrovector mediates effective in vivo suicide gene delivery in experimental brain cancer. Cancer Res. 1999;59:2384-94.

42. Girald W, Collin A, Izquierdo M. Toxicity and delivery methods for the linamarase/linamarin/glucose oxidase system, when used against human glioma tumors implanted in the brain of nude rats. Cancer Letters. 2011;313:99-107.

43. Glick RP, Lichtor T, De ZE, Deshmukh P, Cohen EP. Prolongation of survival of mice with glioma treated with semiallogeneic fibroblasts secreting interleukin-2. Neurosurgery. 1999;45:867-74.

44. Glick RP, Lichtor T, Lin H, Tarlock K, Cohen EP. Immunogene therapy as a treatment for malignant brain tumors in young mice. Journal of Neurosurgery. 2006;105:65-70.

45. Glick RP, Lichtor T, Mogharbel A, Taylor CA, Cohen EP. Intracerebral versus subcutaneous immunization with allogeneic fibroblasts genetically engineered to secrete interleukin-2 in the treatment of central nervous system glioma and melanoma. Neurosurgery41(4)()(pp 898-907), 1997Date of Publication: Oct 1997. 1997:898-907.

46. Goldman CK, Kendall RL, Cabrera G, Soroceanu L, Heike Y, Gillespie GY, et al. Paracrine expression of a native soluble vascular endothelial growth factor receptor inhibits tumor growth, metastasis, and mortality rate. Proc NatlAcadSci US A. 1998;95:8795-800.

47. Gomez-Manzano C, Balague C, Alemany R, Lemoine MG, Mitlianga P, Jiang H, et al. A novel E1A-E1B mutant adenovirus induces glioma regression in vivo. Oncogene. 2004;23:1821-8.

48. Hamed HA, Yacoub A, Park MA, Eulitt PJ, Dash R, Sarkar D, et al. Inhibition of Multiple Protective Signaling Pathways and Ad.5/3 Delivery Enhances mda-7/IL-24 Therapy of Malignant Glioma. Molecular Therapy. 2010;18:1130-42.

49. Harada K, Yoshida J, Mizuno M, Kurisu K, Uozumi T. Growth inhibition of intracerebral rat glioma by transfection-induced human interferon-beta. Journal of Surgical Oncology59(2)()(pp 105-109), 1995Date of Publication: 1995. 1995:105-9.

50. Harding TC, Lalani AS, Roberts BN, Yendluri S, Luan B, Koprivnikar KE, et al. AAV serotype 8-mediated gene delivery of a soluble VEGF receptor to the CNS for the treatment of glioblastoma. MolTher. 2006;13:956-66.

51. Hasegawa Y, Kinoh H, Iwadate Y, Onimaru M, Ueda Y, Harada Y, et al. Urokinase-targeted fusion by oncolytic Sendai virus eradicates orthotopic glioblastomas by pronounced synergy with interferon-beta gene. MolTher. 2010;18:1778-86.

52. Hellums EK, Markert JM, Parker JN, He B, Perbal B, Roizman B, et al. Increased efficacy of an interleukin-12-secreting herpes simplex virus in a syngeneic intracranial murine glioma model. Neuro Oncol. 2005;7:213-24.

53. Herrlinger U, Kramm CM, boody-Guterman KS, Silver JS, Ikeda K, Johnston KM, et al. Pre-existing herpes simplex virus 1 (HSV-1) immunity decreases, but does not abolish, gene transfer to experimental brain tumors by a HSV-1 vector. Gene Ther. 1998;5:809-19.

54. Herrlinger U, Kramm CM, Johnston KM, Louis DN, Finkelstein D, Reznikoff G, et al. Vaccination for experimental gliomas using GM-CSF-transduced glioma cells. Cancer Gene Ther. 1997;4:345-52.

55. Hoffmann D, Meyer B, Wildner O. Improved glioblastoma treatment with Ad5/35 fiber chimeric conditionally replicating adenoviruses. J Gene Med. 2007;9:764-78.

56. Huang Q, Liu XZ, Kang CS, Wang GX, Zhong Y, Pu PY. The anti-glioma effect of suicide gene therapy using BMSC expressing HSV/TK combined with overexpression of Cx43 in glioma cells. Cancer Gene Ther. 2010;17:192-202.

57. Huang Q, Pu P, Xia Z, You Y. Exogenous wt-p53 enhances the antitumor effect of HSV-TK/GCV on C6 glioma cells. J Neurooncol. 2007;82:239-48.

58. Huang Q, Xia Z, You Y, Pu P. Wild Type p53 gene sensitizes rat C6 glioma cells to HSV-TK/ACV treatment in vitro and in vivo. PatholOncolRes. 2010;16:509-14.

59. Huang S, Li J, Han L, Liu S, Ma H, Huang R, et al. Dual targeting effect of Angiopep-2-modified, DNA-loaded nanoparticles for glioma. Biomaterials. 2011;32:6832-8.

60. Huszthy PC, Brekken C, Pedersen TB, Thorsen F, Sakariassen PO, Skaftnesmo KO, et

al. Antitumor efficacy improved by local delivery of species-specific endostatin. Journal of Neurosurgery104(1)()(pp 118-128), 2006Date of Publication: Jan 2006. 2006:118-28.

61. Huszthy PC, Giroglou T, Tsinkalovsky O, Euskirchen P, Skaftnesmo KO, Bjerkvig R, et al. Remission of invasive, cancer stem-like glioblastoma xenografts using lentiviral vector-mediated suicide gene therapy. PLoSOne. 2009;4:e6314.

62. Huszthy PC, Goplen D, Thorsen F, Immervoll H, Wang J, Gutermann A, et al. Oncolytic herpes simplex virus type-1 therapy in a highly infiltrative animal model of human glioblastoma. Clinical Cancer Research. 2008;14:1571-80.

63. Ikeda K, Wakimoto H, Ichikawa T, Jhung S, Hochberg FH, Louis DN, et al. Complement depletion facilitates the infection of multiple brain tumors by an intravascular, replication-conditional herpes simplex virus mutant. J Virol. 2000;74:4765-75.

64. Ito S, Natsume A, Shimato S, Ohno M, Kato T, Chansakul P, et al. Human neural stem cells transduced with IFN-beta and cytosine deaminase genes intensify bystander effect in experimental glioma. Cancer Gene Ther. 2010;17:299-306.

65. Ito S, Natsume A, Shimato S, Ohno M, Kato T, Chansakul P, et al. Human neural stem cells transduced with IFN-beta and cytosine deaminase genes intensify bystander effect in experimental glioma. Cancer Gene Ther. 2010;17:299-306.

66. Iwadate Y, Inoue M, Saegusa T, Tokusumi Y, Kinoh H, Hasegawa M, et al. Recombinant Sendai virus vector induces complete remission of established brain tumors through efficient Interleukin-2 gene transfer in vaccinated rats. Clinical Cancer Research11(10)()(pp 3821-3827), 2005Date of Publication: 15 May 2005. 2005:3821-7.

67. Iwadate Y, Tagawa M, Namba H, Oga M, Kawamura K, Tasaki K, et al. Immunological responsiveness to interleukin-2-producing brain tumors can be restored by concurrent subcutaneous transplantation of the same tumors. Cancer Gene Therapy7(9)()(pp 1263-1269), 2000Date of Publication: 2000. 2000:1263-9.

68. Jeong M, Kwon YS, Park SH, Kim CY, Jeun SS, Song KW, et al. Possible novel therapy for malignant gliomas with secretable trimeric TRAIL. PLoS One. 2009;4:e4545.

69. Jia Q, Li Y, Xu D, Li Z, Zhang Z, Zhang Y, et al. Radiosensitivity of glioma to Gamma Knife treatment enhanced in vitro and in vivo by RNA interfering Ku70 that is mediated by a recombinant adenovirus. JNeurosurg. 2010;113 Suppl:228-35.

70. Kanai R, Rabkin SD, Yip S, Sgubin D, Zaupa CM, Hirose Y, et al. Oncolytic virus-mediated manipulation of DNA damage responses: synergy with chemotherapy in killing glioblastoma stem cells. JNatlCancer Inst. 2012;104:42-55.

71. Kanai R, Tomita H, Shinoda A, Takahashi M, Goldman S, Okano H, et al. Enhanced therapeutic efficacy of G207 for the treatment of glioma through Musashi1 promoter retargeting of gamma34.5-mediated virulence. Gene Ther. 2006;13:106-16.

72. Kanai R, Zaupa C, Sgubin D, Antoszczyk SJ, Martuza RL, Wakimoto H, et al. Effect of

gamma34.5 deletions on oncolytic herpes simplex virus activity in brain tumors. JVirol. 2012;86:4420-31.

73. Kato T, Natsume A, Toda H, Iwamizu H, Sugita T, Hachisu R, et al. Efficient delivery of liposome-mediated MGMT-siRNA reinforces the cytotoxity of temozolomide in GBM-initiating cells. Gene Therapy. 2010;17:1363-71.

74. Kikuchi T, Joki T, Akasaki Y, Abe T, Ohno T. Antitumor activity of interleukin 12 against interleukin 2-transduced mouse glioma cells. Cancer Letters. 1999;135:47-51.

75. Kim CH, Hong MJ, Park SD, Kim CK, Park MY, Sohn HJ, et al. Enhancement of anti-tumor immunity specific to murine glioma by vaccination with tumor cell lysate-pulsed dendritic cells engineered to produce interleukin-12. Cancer ImmunolImmunother. 2006;55:1309-19.

76. Kim CH, Woo SJ, Park JS, Kim HS, Park MY, Park SD, et al. Enhanced antitumour immunity by combined use of temozolomide and TAT-survivin pulsed dendritic cells in a murine glioma. Immunology. 2007;122:615-22.

77. Kim SM, Lim JY, Park SI, Jeong CH, Oh JH, Jeong M, et al. Gene therapy using TRAIL-secreting human umbilical cord blood-derived mesenchymal stem cells against intracranial glioma. Cancer Res. 2008;68:9614-23.

78. Kurozumi K, Hardcastle J, Thakur R, Yang M, Christoforidis G, Fulci G, et al. Effect of tumor microenvironment modulation on the efficacy of oncolytic virus therapy. J NatlCancer Inst. 2007;99:1768-81.

79. Kuwashima N, Nishimura F, Eguchi J, Sato H, Hatano M, Tsugawa T, et al. Delivery of dendritic cells engineered to secrete IFN-alpha into central nervous system tumors enhances the efficacy of peripheral tumor cell vaccines: dependence on apoptotic pathways. J Immunol. 2005;175:2730-40.

80. Lal B, Xia S, Abounader R, Laterra J. Targeting the c-Met pathway potentiates glioblastoma responses to gamma-radiation. Clin Cancer Res. 2005;11:4479-86.

81. Lamfers ML, Gianni D, Tung CH, Idema S, Schagen FH, Carette JE, et al. Tissue inhibitor of metalloproteinase-3 expression from an oncolytic adenovirus inhibits matrix metalloproteinase activity in vivo without affecting antitumor efficacy in malignant glioma. Cancer Res. 2005;65:9398-405.

82. Lau CJ, Koty Z, Nalbantoglu J. Differential response of glioma cells to FOXO1-directed therapy. Cancer Res. 2009;69:5433-40.

83. Lee EX, Lam DH, Wu C, Yang J, Tham CK, Ng WH, et al. Glioma gene therapy using induced pluripotent stem cell derived neural stem cells. MolPharm. 2011;8:1515-24.

84. Lee SJ, Kim SJ, Seo HH, Shin SP, Kim D, Park CS, et al. Over-expression of miR-145 enhances the effectiveness of HSVtk gene therapy for malignant glioma. Cancer Lett. 2012;320:72-80.

85. Li CH, Jiao BH. [Effect of bone marrow stromal cells transfected with interleukin 18 on growth of intracranial glioma in rats]. AiZheng. 2007;26:38-43.

86. Li H, onso-Vanegas M, Colicos MA, Jung SS, Lochmuller H, Sadikot AF, et al. Intracerebral adenovirus-mediated p53 tumor suppressor gene therapy for experimental human glioma. Clin Cancer Res. 1999;5:637-42.

87. Li J, Gu B, Meng Q, Yan Z, Gao H, Chen X, et al. The use of myristic acid as a ligand of polyethylenimine/DNA nanoparticles for targeted gene therapy of glioblastoma. Nanotechnology. 2011;22:435101.

88. Li S, Tokuyama T, Yamamoto J, Koide M, Yokota N, Namba H. Bystander effect-mediated gene therapy of gliomas using genetically engineered neural stem cells. Cancer Gene Therapy12(7)()(pp 600-607), 2005Date of Publication: Jul 2005. 2005:600-7.

89. Liang B, He ML, Chan C, Chen Y, Li XP, Li Y, et al. The use of folate-PEG-grafted-hybranched-PEI nonviral vector for the inhibition of glioma growth in the rat. Biomaterials30(23-24)()(pp 4014-4020), 2009Date of Publication: August 2009. 2009:4014-20.

90. Liau LM, Fakhrai H, Black KL. Prolonged survival of rats with intracranial C6 gliomas by treatment with TGF-beta antisense gene. Neurological Research20(8)()(pp 742-747), 1998Date of Publication: Dec 1998. 1998:742-7.

91. Lichtor T, Glick RP. Cytokine immuno-gene therapy for treatment of brain tumors. Journal of Neuro-Oncology65(3)()(pp 247-259), 2003Date of Publication: Dec 2003. 2003:247-59.

92. Lichtor T, Glick RP, Tae SK, Hand R, Cohen EP. Prolonged survival of mice with glioma injected intracerebrally with double cytokine-secreting cells. Journal of Neurosurgery83(6)()(pp 1038-1044), 1995Date of Publication: 1995. 1995:1038-44.

93. Lichtor T, Glick RP, Tarlock K, Moffett S, Mouw E, Cohen EP. Application of interleukin-2-secreting syngeneic/allogeneic fibroblasts in the treatment of primary and metastatic brain tumors. Cancer Gene Therapy9(5)()(pp 464-469), 2002Date of Publication: 2002. 2002:464-9.

94. Liu S, Guo Y, Huang R, Li J, Huang S, Kuang Y, et al. Gene and doxorubicin co-delivery system for targeting therapy of glioma. Biomaterials. 2012;33:June.

95. Liu Y, Ehtesham M, Samoto K, Wheeler CJ, Thompson RC, Villarreal LP, et al. In situ adenoviral interleukin 12 gene transfer confers potent and long-lasting cytotoxic immunity in glioma. Cancer Gene Ther. 2002;9:9-15.

96. Liu Y, Lang F, Xie X, Prabhu S, Xu J, Sampath D, et al. Efficacy of adenovirally expressed soluble TRAIL in human glioma organotypic slice culture and glioma xenografts. Cell Death Dis. 2011;2:e121.

97. Lu W, Sun Q, Wan J, She Z, Jiang XG. Cationic albumin-conjugated pegylated nanoparticles allow gene delivery into brain tumors via intravenous administration. Cancer Research66(24)()(pp 11878-11887), 2006Date of Publication: 15 Dec 2006. 2006:11878-87.

98. Lumniczky K, Desaknai S, Mangel L, Szende B, Hamada H, Hidvegi EJ, et al. Local tumor irradiation augments the antitumor effect of cytokine-producing autologous cancer cell vaccines in a murine glioma model. Cancer Gene Therapy9(1)()(pp 44-52), 2002Date of Publication: 2002. 2002:44-52.

99. Lun X, Senger DL, Alain T, Oprea A, Parato K, Stojdl D, et al. Effects of intravenously administered recombinant vesicular stomatitis virus (VSV(deltaM51)) on multifocal and invasive gliomas. J NatlCancer Inst. 2006;98:1546-57.

100. Lun XQ, Jang JH, Tang N, Deng H, Head R, Bell JC, et al. Efficacy of systemically administered oncolytic vaccinia virotherapy for malignant gliomas is enhanced by combination therapy with rapamycin or cyclophosphamide. Clin Cancer Res. 2009;15:2777-88.

101. Ma HI, Lin SZ, Chiang YH, Li J, Chen SL, Tsao YP, et al. Intratumoral gene therapy of malignant brain tumor in a rat model with angiostatin delivered by adeno-associated viral (AAV) vector. Gene Therapy9(1)()(pp 2-11), 2002Date of Publication: 2002. 2002:2-11.

102. Machein MR, Risau W, Plate KH. Antiangiogenic gene therapy in a rat glioma model using a dominant-negative vascular endothelial growth factor receptor 2. Human Gene Therapy10(7)()(pp 1117-1128), 1999Date of Publication: 01 May 1999. 1999:1117-28.

103. Maeda M, Namikawa K, Kobayashi I, Ohba N, Takahara Y, Kadono C, et al. Targeted gene therapy toward astrocytoma using a Cre/loxP-based adenovirus system. Brain Research1081(1)()(pp 34-43), 2006Date of Publication: 07 Apr 2006. 2006:34-43.

104. Maguire CA, Meijer DH, LeRoy SG, Tierney LA, Broekman ML, Costa FF, et al. Preventing growth of brain tumors by creating a zone of resistance. MolTher. 2008;16:1695-702.

105. Manome Y, Wen PY, Chen L, Tanaka T, Dong Y, Yamazoe M, et al. Gene therapy for malignant gliomas using replication incompetent retroviral and adenoviral vectors encoding the cytochrome P450 2B1 gene together with cyclophosphamide. Gene Ther. 1996;3:513-20.

106. Marconi P, Tamura M, Moriuchi S, Krisky DM, Niranjan A, Goins WF, et al. Connexin 43-enhanced suicide gene therapy using herpesviral vectors. MolTher. 2000;1:71-81.

107. Markert JM, Cody JJ, Parker JN, Coleman JM, Price KH, Kern ER, et al. Preclinical evaluation of a genetically engineered herpes simplex virus expressing interleukin-12. JVirol. 2012;86:5304-13.

108. Maron A, Gustin T, Le RA, Mottet I, Dedieu JF, Brion JP, et al. Gene therapy of rat C6 glioma using adenovirus-mediated transfer of the herpes simplex virus thymidine kinase gene: Long-term follow-up by magnetic resonance imaging. Gene Therapy3(4)()(pp 315-322), 1996Date of Publication: Apr 1996. 1996:315-22.

109. Matsuda M, Nimura K, Shimbo T, Hamasaki T, Yamamoto T, Matsumura A, et al. Immunogene therapy using immunomodulating HVJ-E vector augments anti-tumor effects in murine malignant glioma. JNeurooncol. 2011;103:19-31.

110. Matsuda M, Yamamoto T, Matsumura A, Kaneda Y. Highly efficient eradication of intracranial glioblastoma using Eg5 siRNA combined with HVJ envelope. Gene Ther. 2009;16:1465-76.

111. Meijer DH, Maguire CA, LeRoy SG, Sena-Esteves M. Controlling brain tumor growth by intraventricular administration of an AAV vector encoding IFN-beta. Cancer Gene Ther. 2009;16:664-71.

112. Mineta T, Rabkin SD, Yazaki T, Hunter WD, Martuza RL. Attenuated multi-mutated herpes simplex virus-1 for the treatment of malignant gliomas. NatMed. 1995;1:938-43.

113. Miura F, Moriuchi S, Maeda M, Sano A, Maruno M, Tsanaclis AM, et al. Sustained release of low-dose ganciclovir from a silicone formulation prolonged the survival of rats with gliosarcomas under herpes simplex virus thymidine kinase suicide gene therapy. Gene Therapy9(24)()(pp 1653-1658), 2002Date of Publication: Dec 2002. 2002:1653-8.

114. Mori K, Iwata J, Miyazaki M, Osada H, Tange Y, Yamamoto T, et al. Bystander killing effect of tymidine kinase gene-transduced adult bone marrow stromal cells with ganciclovir on malignant glioma cells. Neurologia Medico-Chirurgica50 (7) ()(pp 545-553), 2010Date of Publication: 2010. 2010:545-53.

115. Mori K, Iwata J, Miyazaki M, Osada H, Tange Y, Yamamoto T, et al. Bystander killing effect of tymidine kinase gene-transduced adult bone marrow stromal cells with ganciclovir on malignant glioma cells. NeurolMedChir (Tokyo). 2010;50:545-53.

116. Morioka J, Kajiwara K, Yoshikawa K, Ideguchi M, Uchida T, Ohmoto Y, et al. Adenovirus-mediated gene transfer of B7.1 induces immunological anti-tumor effects in a murine brain tumor. Journal of Neuro-Oncology60(1)()(pp 13-23), 2002Date of Publication: 01 Oct 2002. 2002:13-23.

117. Moriuchi S, Glorioso JC, Maruno M, Izumoto S, Wolfe D, Huang S, et al. Combination gene therapy for glioblastoma involving herpes simplex virus vector-mediated codelivery of mutant IkappaBalpha and HSV thymidine kinase. Cancer Gene Therapy12(5)()(pp 487-496), 2005Date of Publication: May 2005. 2005:487-96.

118. Moriuchi S, Krisky DM, Marconi PC, Tamura M, Shimizu K, Yoshimine T, et al. HSV vector cytotoxicity is inversely correlated with effective TK/GCV suicide gene therapy of rat gliosarcoma. Gene Therapy7(17)()(pp 1483-1490), 2000Date of Publication: 2000. 2000:1483-90.

119. Moriuchi S, Oligino T, Krisky D, Marconi P, Fink D, Cohen J, et al. Enhanced tumor cell killing in the presence of ganciclovir by herpes simplex virus type 1 vector-directed coexpression of human tumor necrosis factor-alpha and herpes simplex virus thymidine kinase. Cancer Res. 1998;58:5731-7.

120. Nafe C, Cao YJ, Quinones A, Dobberstein KU, Kramm CM, Rainov NG. Expression of mutant non-cleavable Fas ligand on retrovirus packaging cells causes apoptosis of immunocompetent cells and improves prodrug activation gene therapy in a malignant glioma

model. Life Sci. 2003;73:1847-60.

121. Nam M, Johnston P, Lal B, Indurti R, Wilson MA, Laterra J. Endothelial cell-based cytokine gene delivery inhibits 9L glioma growth in vivo. Brain Research731(1-2)()(pp 161-170), 1996Date of Publication: 26 Aug 1996. 1996:161-70.

122. Namba H, Tagawa M, Iwadate Y, Kimura M, Sueyoshi K, Sakiyama S. Bystander effect-mediated therapy of experimental brain tumor by genetically engineered tumor cells. Human Gene Therapy9(1)()(pp 5-11), 1998Date of Publication: 01 Jan 1998. 1998:5-11.

123. Natsume A, Mizuno M, Ryuke Y, Yoshida J. Antitumor effect and cellular immunity activation by murine interferon-beta gene transfer against intracerebral glioma in mouse. Gene Ther. 1999;6:1626-33.

124. Nestler U, Wakimoto H, Siller-Lopez F, Aguilar LK, Chakravarti A, Muzikansky A, et al. The combination of adenoviral HSV TK gene therapy and radiation is effective in athymic mouse glioblastoma xenografts without increasing toxic side effects. J Neurooncol. 2004;67:177-88.

125. Niranjan A, Moriuchi S, Lunsford LD, Kondziolka D, Flickinger JC, Fellows W, et al. Effective treatment of experimental glioblastoma by HSV vector-mediated TNF alpha and HSV-tk gene transfer in combination with radiosurgery and ganciclovir administration. MolTher. 2000;2:114-20.

126. Oh S, Ohlfest JR, Todhunter DA, Vallera VD, Hall WA, Chen H, et al. Intracranial elimination of human glioblastoma brain tumors in nude rats using the bispecific ligand-directed toxin, DTEGF13 and convection enhanced delivery. J Neurooncol. 2009;95:331-42.

127. Ohlfest JR, Demorest ZL, Motooka Y, Vengco I, Oh S, Chen E, et al. Combinatorial antiangiogenic gene therapy by nonviral gene transfer using the sleeping beauty transposon causes tumor regression and improves survival in mice bearing intracranial human glioblastoma. MolTher. 2005;12:778-88.

128. Okada T, Shah M, Higginbotham JN, Li Q, Wildner O, Walbridge S, et al. AV.TK-mediated killing of subcutaneous tumors in situ results in effective immunization against established secondary intracranial tumor deposits. Gene Ther. 2001;8:1315-22.

129. Otsuki A, Patel A, Kasai K, Suzuki M, Kurozumi K, Chiocca EA, et al. Histone deacetylase inhibitors augment antitumor efficacy of herpes-based oncolytic viruses. MolTher. 2008;16:1546-55.

130. Paraskevakou G, Allen C, Nakamura T, Zollman P, James CD, Peng KW, et al. Epidermal growth factor receptor (EGFR)-Retargeted measles virus strains effectively target EGFR- or EGFRvIII expressing gliomas. Molecular Therapy15(4)()(pp 677-686), 2007Date of Publication: Apr 2007. 2007:677-86.

131. Paul DB, Barth RF, Yang W, Shen GH, Kim J, Triozzi PL. B7.1 expression by the weakly immunogenic F98 rat glioma does not enhance immunogenicity. Gene Ther. 2000;7:993-9.

132. Pellegatta S, Poliani PL, Corno D, Menghi F, Ghielmetti F, Suarez-Merino B, et al.

Neurospheres enriched in cancer stem-like cells are highly effective in eliciting a dendritic cell-mediated immune response against malignant gliomas. Cancer Res. 2006;66:10247-52.

133. Peres EA, Valable S, Guillamo JS, Marteau L, Bernaudin JF, Roussel S, et al. Targeting the erythropoietin receptor on glioma cells reduces tumour growth. ExpCell Res. 2011;317:2321-32.

134. Perri SR, Nalbantoglu J, Annabi B, Koty Z, Lejeune L, Francois M, et al. Plasminogen kringle 5-engineered glioma cells block migration of tumor-associated macrophages and suppress tumor vascularization and progression. Cancer Res. 2005;65:8359-65.

135. Persson BRR, Koch CB, Grafstrm G, Ceberg C, Rosenschld PM, Nittby H, et al. Radiation immunomodulatory gene tumor therapy of rats with intracerebral glioma tumors. Radiation Research173(4)()(pp 433-440), 2010Date of Publication: April 2010. 2010:433-40.

136. Phuong LK, Allen C, Peng KW, Giannini C, Greiner S, TenEyck CJ, et al. Use of a vaccine strain of measles virus genetically engineered to produce carcinoembryonic antigen as a novel therapeutic agent against glioblastoma multiforme. Cancer Res. 2003;63:2462-9.

137. Redaelli M, Franceschi V, Capocefalo A, D'Avella D, Denaro L, Cavirani S, et al. Herpes simplex virus type 1 thymidine kinase-armed bovine herpesvirus type 4-based vector displays enhanced oncolytic properties in immunocompetent orthotopic syngenic mouse and rat glioma models. Neuro Oncol. 2012;14:288-301.

138. Roche FP, Sheahan BJ, O'Mara SM, Atkins GJ. Semliki Forest virus-mediated gene therapy of the RG2 rat glioma. Neuropathology and Applied Neurobiology36 (7) ()(pp 648-660), 2010Date of Publication: December 2010. 2010:648-60.

139. Ross BD, Kim B, Davidson BL. Assessment of ganciclovir toxicity to experimental intracranial gliomas following recombinant adenoviral-mediated transfer of the herpes simplex virus thymidine kinase gene by magnetic resonance imaging and proton magnetic resonance spectroscopy. Clinical Cancer Research1(6)()(pp 651-657), 1995Date of Publication: 1995. 1995:651-7.

140. Ryu CH, Park SH, Park SA, Kim SM, Lim JY, Jeong CH, et al. Gene therapy of intracranial glioma using interleukin 12-secreting human umbilical cord blood-derived mesenchymal stem cells. HumGene Ther. 2011;22:733-43.

141. Saka M, Amano T, Kajiwara K, Yoshikawa K, Ideguchi M, Nomura S, et al. Vaccine therapy with dendritic cells transfected with Il13ra2 mRNA for glioma in mice. J Neurosurg. 2010;113:270-9.

142. Samoto K, Ehtesham M, Perng GC, Hashizume K, Wechsler SL, Nesburn AB, et al. A herpes simplex virus type 1 mutant with gamma 34.5 and LAT deletions effectively oncolyses human U87 glioblastomas in nude mice. Neurosurgery. 2002;50:599-605.

143. Sandmair AM, Turunen M, Tyynela K, Loimas S, Vainio P, Vanninen R, et al. Herpes simplex virus thymidine kinase gene therapy in experimental rat BT4C glioma model: Effect of

the percentage of thymidine kinase-positive glioma cells on treatment effect, survival time, and tissue reactions. Cancer Gene Therapy7(3)()(pp 413-421), 2000Date of Publication: 2000. 2000:413-21.

144. Santra M, Zheng X, Roberts C, Santra S, Lu M, Panda S, et al. Single doublecortin gene therapy significantly reduces glioma tumor volume. J NeurosciRes. 2010;88:304-14.

145. Sato H, Kuwashima N, Sakaida T, Hatano M, Dusak JE, Fellows-Mayle WK, et al. Epidermal growth factor receptor-transfected bone marrow stromal cells exhibit enhanced migratory response and therapeutic potential against murine brain tumors. Cancer Gene Therapy12(9)()(pp 757-768), 2005Date of Publication: Sep 2005. 2005:757-68.

146. Schneider T, Becker A, Ringe K, Reinhold A, Firsching R, Sabel BA. Brain tumor therapy by combined vaccination and antisense oligonucleotide delivery with nanoparticles. J Neuroimmunol. 2008;195:21-7.

147. Shah AC, Parker JN, Gillespie GY, Lakeman FD, Meleth S, Markert JM, et al. Enhanced antiglioma activity of chimeric HCMV/HSV-1 oncolytic viruses. Gene Ther. 2007;14:1045-54.

148. Shah AC, Price KH, Parker JN, Samuel SL, Meleth S, Cassady KA, et al. Serial passage through human glioma xenografts selects for a Deltagamma134.5 herpes simplex virus type 1 mutant that exhibits decreased neurotoxicity and prolongs survival of mice with experimental brain tumors. J Virol. 2006;80:7308-15.

149. Shir A, Ogris M, Wagner E, Levitzki A. EGF receptor-targeted synthetic double-stranded RNA eliminates glioblastoma, breast cancer, and adenocarcinoma tumors in mice. Plos Medicine. 2006;3:125-35.

150. Sonabend AM, Velicu S, Ulasov IV, Han Y, Tyler B, Brem H, et al. A safety and efficacy study of local delivery of interleukin-12 transgene by PPC polymer in a model of experimental glioma. Anti-Cancer Drugs19(2)()(pp 133-142), 2008Date of Publication: Feb 2008. 2008:133-42.

151. Sun X, Pang Z, Ye H, Qiu B, Guo L, Li J, et al. Co-delivery of pEGFP-hTRAIL and paclitaxel to brain glioma mediated by an angiopep-conjugated liposome. Biomaterials. 2012;33:916-24.

152. Szatmari T, Huszty G, Desaknai S, Spasokoukotskaja T, Sasvari-Szekely M, Staub M, et al. Adenoviral vector transduction of the human deoxycytidine kinase gene enhances the cytotoxic and radiosensitizing effect of gemcitabine on experimental gliomas. Cancer Gene Ther. 2008;15:154-64.

153. Tabatabai G, Hasenbach K, Herrmann C, Maurer G, Mohle R, Marini P, et al. Glioma tropism of lentivirally transduced hematopoietic progenitor cells. Int J Oncol. 2010;36:1409-17.

154. Tamura K, Tamura M, Ikenaka K, Yoshimatsu T, Miyao Y, Nanmoku K, et al. Eradication of murine brain tumors by direct inoculation of concentrated high titer-recombinat retrovirus harboring the herpes simplex virus thymidine kinase gene. Gene Therapy8(3)()(pp 215-222),

2001Date of Publication: 2001. 2001:215-22.

155. Tse V, Yung Y, Santarelli JG, Juan D, Hsiao M, Haas M, et al. Effects of Tumor Suppressor Gene (p53) on Brain Tumor Angiogenesis and Expression of Angiogenic Modulators. Anticancer Research24(1)()(pp 1-10), 2004Date of Publication: Jan 2004. 2004:1-10.

156. Tseng SH, Hwang LH, Lin SM. Induction of antitumor immunity by intracerebrally implanted rat C6 glioma cells genetically engineered to secrete cytokines. J Immunother. 1997;20:334-42.

157. Tsugawa T, Kuwashima N, Sato H, Fellows-Mayle WK, Dusak JE, Okada K, et al. Sequential delivery of interferon-alpha gene and DCs to intracranial gliomas promotes an effective antitumor response. Gene Ther. 2004;11:1551-8.

158. Ueda R, Kinoshita E, Ito R, Kawase T, Kawakami Y, Toda M. Induction of protective and therapeutic antitumor immunity by a DNA vaccine with a glioma antigen, SOX6. International Journal of Cancer. 2008;122:2274-9.

159. Ulasov IV, Rivera AA, Nettelbeck DM, Rivera LB, Mathis JM, Sonabend AM, et al. An oncolytic adenoviral vector carrying the tyrosinase promoter for glioma gene therapy. International Journal of Oncology. 2007;31:1177-85.

160. Ulasov IV, Zhu ZB, Tyler MA, Han Y, Rivera AA, Khramtsov A, et al. Survivin-driven and fiber-modified oncolytic adenovirus exhibits potent antitumor activity in established intracranial glioma. HumGene Ther. 2007;18:589-602.

161. Varma NR, Janic B, Iskander AS, Shankar A, Bhuiyan MP, Soltanian-Zadeh H, et al. Endothelial progenitor cells (EPCs) as gene carrier system for rat model of human glioma. PLoS One. 2012;7:e30310.

162. Verheije MH, Lamfers MLM, Wurdinger T, Grinwis GCM, Gerritsen WR, van Beusechem VW, et al. Coronavirus Genetically Redirected to the Epidermal Growth Factor Receptor Exhibits Effective Antitumor Activity against a Malignant Glioblastoma. Journal of Virology. 2009;83:7507-16.

163. Vincent AJ, Esandi MC, Avezaat CJ, Vecht CJ, Sillevis SP, van Bekkum DW, et al. Preclinical testing of recombinant adenoviral herpes simplex virus-thymidine kinase gene therapy for central nervous system malignancies. Neurosurgery. 1997;41:442-51.

164. Vincent AJ, Vogels R, Someren GV, Esandi MC, Noteboom JL, Avezaat CJ, et al. Herpes simplex virus thymidine kinase gene therapy for rat malignant brain tumors. HumGene Ther. 1996;7:197-205.

165. Visse E, Siesjo P, Widegren B, Sjogren HO. Regression of intracerebral rat glioma isografts by therapeutic subcutaneous immunization with interferon-gamma, interleukin-7, or B7-1-transfected tumor cells. Cancer Gene Ther. 1999;6:37-44.

166. Wang F, Bai HR, Wang J, Bai YZ, Dou CW. Glioma growth inhibition in vitro and in vivo

by single chain variable fragments of the transferrin receptor conjugated to survivin small interfering RNA. JIntMedRes. 2011;39:1701-12.

167. Wang TJ, Huang MS, Hong CY, Tse V, Silverberg GD, Hsiao M. Comparisons of tumor suppressor p53, p21, and p16 gene therapy effects on glioblastoma tumorigenicity in Situ. Biochemical and Biophysical Research Communications287(1)()(pp 173-180), 2001Date of Publication: 14 Sep 2001. 2001:173-80.

168. Wang ZH, Zagzag D, Zeng B, Kolodny EH. In vivo and in vitro glioma cell killing induced by an adenovirus expressing both cytosine deaminase and thymidine kinase and its association with interferon-alpha. Journal of Neuropathology and Experimental Neurology58(8)()(pp 847-858), 1999Date of Publication: Aug 1999. 1999:847-58.

169. Wei MX, Tamiya T, Hurford RK, Jr., Boviatsis EJ, Tepper RI, Chiocca EA. Enhancement of interleukin-4-mediated tumor regression in athymic mice by in situ retroviral gene transfer. HumGene Ther. 1995;6:437-43.

170. Wirth T, Pikkarainen JT, Samaranayake HD, Lehtolainen-Dalkilic P, Lesch HP, Airenne KJ, et al. Efficient gene therapy based targeting system for the treatment of inoperable tumors. Journal of Gene Medicine. 2012;14:April.

171. Wu A, Oh S, Ericson K, Demorest ZL, Vengco I, Gharagozlou S, et al. Transposon-based interferon gamma gene transfer overcomes limitations of episomal plasmid for immunogene therapy of glioblastoma. Cancer Gene Therapy14(6)()(pp 550-560), 2007Date of Publication: Jun 2007. 2007:550-60.

172. Xu D, Jia Q, Li Y, Kang C, Pu P. Effects of Gamma Knife surgery on C6 glioma in combination with adenoviral p53 in vitro and in vivo. J Neurosurg. 2006;105 Suppl:208-13.

173. Yacoub A, Hamed H, Emdad L, Dos SW, Gupta P, Broaddus WC, et al. MDA-7/IL-24 plus radiation enhance survival in animals with intracranial primary human GBM tumors. Cancer Biol Ther. 2008;7:917-33.

174. Yacoub A, Mitchell C, Lister A, Lebedeva IV, Sarkar D, Su ZZ, et al. Melanoma differentiation-associated 7 (interleukin 24) inhibits growth and enhances radiosensitivity of glioma cells in vitro and in vivo. Clin Cancer Res. 2003;9:3272-81.

175. Yamanaka R, Tanaka R, Yoshida S, Saitoh T, Fujita K. Growth inhibition of human glioma cells modulated by retrovirus gene transfection with antisense IL-8. J Neurooncol. 1995;25:59-65.

176. Yamanaka R, Tanaka R, Yoshida S, Saitoh T, Fujita K, Naganuma H. Suppression of TGF-beta1 in human gliomas by retroviral gene transfection enhances susceptibility to LAK cells. J Neurooncol. 1999;43:27-34.

177. Yamanaka R, Yajima N, Tsuchiya N, Honma J, Tanaka R, Ramsey J, et al. Administration of interleukin-12 and -18 enhancing the antitumor immunity of genetically modified dendritic cells that had been pulsed with Semliki forest virus-mediated tumor

complementary DNA. J Neurosurg. 2002;97:1184-90.

178. Yamanaka R, Zullo SA, Tanaka R, Blaese M, Xanthopoulos KG. Enhancement of antitumor immune response in glioma models in mice by genetically modified dendritic cells pulsed with Semliki Forest virus-mediated complementary DNA. Journal of Neurosurgery94(3)()(pp 474-481), 2001Date of Publication: 2001. 2001:474-81.

179. Yamanaka R, Zullo SA, Tanaka R, Ramsey J, Blaese M, Xanthopoulos KG. Induction of a therapeutic antitumor immunological response by intratumoral injection of genetically engineered Semliki Forest virus to produce interleukin-12. NeurosurgFocus. 2000;9:e7.

180. Yamini B, Yu X, Gillespie GY, Kufe DW, Weichselbaum RR. Transcriptional targeting of adenovirally delivered tumor necrosis factor alpha by temozolomide in experimental glioblastoma. Cancer Res. 2004;64:6381-4.

181. Yang YP, Chang YL, Huang PI, Chiou GY, Tseng LM, Chiou SH, et al. Resveratrol suppresses tumorigenicity and enhances radiosensitivity in primary glioblastoma tumor initiating cells by inhibiting the STAT3 axis. JCell Physiol. 2012;227:976-93.

182. Yokoyama T, Iwado E, Kondo Y, Aoki H, Hayashi Y, Georgescu MM, et al. Autophagy-inducing agents augment the antitumor effect of telerase-selve oncolytic adenovirus OBP-405 on glioblastoma cells. Gene Ther. 2008;15:1233-9.

183. Yoo JY, Haseley A, Bratasz A, Chiocca EA, Zhang J, Powell K, et al. Antitumor efficacy of 34.5ENVE: a transcriptionally retargeted and "Vstat120"-expressing oncolytic virus. MolTher. 2012;20:287-97.

184. Yoshida J, Mizuno M, Nakahara N, Colosi P. Antitumor effect of an adeno-associated virus vector containing the human interferon-beta gene on experimental intracranial human glioma. JpnJ Cancer Res. 2002;93:223-8.

185. Yoshikawa K, Kajiwara K, Ideguchi M, Uchida T, Ito H. Immune gene therapy of experimental mouse brain tumor with adenovirus- mediated gene transfer of murine interleukin-4. Cancer Immunology Immunotherapy49(1)()(pp 23-33), 2000Date of Publication: 2000. 2000:23-33.

186. Yu JS, Burwick JA, Dranoff G, Breakefield XO. Gene therapy for metastatic brain tumors by vaccination with granulocyte-macrophage colony-stimulating factor-transduced tumor cells. Human Gene Therapy8(9)()(pp 1065-1072), 1997Date of Publication: 10 Jun 1997. 1997:1065-72.

187. Zhang H, Wen YJ, Mao BY, Gong QY, Qian ZY, Wei YQ. Plasmid encoding matrix protein of vesicular stomatitis viruses as an antitumor agent inhibiting rat glioma growth in situ. Experimental Oncology29(2)()(pp 85-93), 2007Date of Publication: Jun 2007. 2007:85-93.

188. Zhang J, Frolov I, Russell SJ. Gene therapy for malignant glioma using Sindbis vectors expressing a fusogenic membrane glycoprotein. Journal of Gene Medicine6(10)()(pp 1082-1091), 2004Date of Publication: Oct 2004. 2004:1082-91.

189. Zhang W, Fulci G, Buhrman JS, Stemmer-Rachamimov AO, Chen JW, Wojtkiewicz GR, et al. Bevacizumab with angiostatin-armed oHSV increases antiangiogenesis and decreases bevacizumab-induced invasion in U87 glioma. MolTher. 2012;20:37-45.

190. Zhang Y, Wang C, Zhang Y, Sun M. C6 glioma cells retrovirally engineered to express IL-18 and Fas exert FasL-dependent cytotoxicity against glioma formation. BiochemBiophysRes Commun. 2004;325:1240-5.

191. Zhang Y, Zhang YF, Bryant J, Charles A, Boado RJ, Pardridge WM. Intravenous RNA interference gene therapy targeting the human epidermal growth factor receptor prolongs survival in intracranial brain cancer. Clin Cancer Res. 2004;10:3667-77.

192. Zhang Y, Zhu C, Pardridge WM. Antisense gene therapy of brain cancer with an artificial virus gene delivery system. Molecular Therapy6(1)()(pp 67-72), 2002Date of Publication: 01 Jul 2002. 2002:67-72.

193. Zhu H, Li JT, Zheng F, Martin E, Kots AY, Krumenacker JS, et al. Restoring soluble guanylyl cyclase expression and function blocks the aggressive course of glioma. MolPharmacol. 2011;80:1076-84.
