# Supplementary material for: A systematic review and meta‐analysis of gene therapy in animal models of cerebral glioma: why did promise not translate to human therapy?
Source: Evid Based Preclin Med. 2015 Jan 20;1(1):e00006. doi: 10.1002/ebm2.6 (PMC5020579; doi:10.1002/ebm2.6)
Supplement: Supplementary file 2 — Appendix S2. Study characteristics. [file EBM2-1-21-s003.pdf]

Supplementary material 2: Study characeteristics

Name	***Year***	***Animal***	***Comorbidity***	***Glioma Model***	***Gene therapy***	***Prodrug***	***Vector***	***Route of delivery***	***Delay to treatment***	***Survival endpoint***
Adachi, Y	2000	Rat	None	9L	CD	5-FC	Adenovirus	Intratumoural	>0	Symptomatic
Adachi, Y	2000	Rat	None	9L	CD and UPRT	5-FC	Adenovirus	Intratumoural	>0	Symptomatic
Aghi, M	2006	Mouse	Athymic	U87	HSV-gamma34.5/RR	None	Adenovirus	Intratumoural	>0	Symptomatic
ahmed, a	2011	Mouse	None	U87	S-pk7	None	Neural stem cell	Intratumoural	>0	Symptomatic
ahmed, a	2011	Mouse	None	U87	S-pk7	None	Adenovirus	Intratumoural	>0	Symptomatic
Ahmed, N	2010	Mouse	SCID	Human GBM	HER2	None	Retrovirus	Intratumoural	>0	Unknown
Ali, S	2005	Rat	None	CNS-1	HSV-fml-TK	None	Adenovirus	Intratumoural	>0	Symptomatic
Ali, S	2005	Rat	None	CNS-1	HSV-fml-TK	None	Adenovirus	Intratumoural	>0	Symptomatic
Allen, C	2008	Mouse	None	GBM12	MV-GFP	None	Retrovirus	Intratumoural	>0	Symptomatic
Allen, C	2008	Mouse	None	GBM12	MV-GFP/HAA-IL-13	None	Retrovirus	Intratumoural	>0	Symptomatic
Alonso, M	2007	Mouse	Athymic	U87	E1A	None	Adenovirus	Intratumoural	>0	Unknown
Alonso, M	2008	Mouse	Athymic	U87	RAD001	None	Adenovirus	Intratumoural	>0	Survival
Altanerova, V	2011	Rat	None	C6	Cdy::UPRT	5-FC	Mesenchymal stem cell	Contralateral	0	Symptomatic

Name	Year	Animal	Comorbidity	Glioma Model	Gene therapy	Prodrug	Vector	Route of delivery	Delay to treatment	Survival endpoint
Altanerova, V	2011	Rat	None	C6	Cdy::UPRT	5-FC	Mesenchymal stem cell	Contralateral	0	Symptomatic
Altanerova, V	2011	Rat	None	C6	Cdy::UPRT	5-FC	Mesenchymal stem cell	Contralateral	0	Symptomatic
Altanerova, V	2011	Rat	None	C6	Cdy::UPRT	5-FC	Mesenchymal stem cell	Contralateral	0	Symptomatic
Altanerova, V	2011	Rat	None	C6	Cdy::UPRT	5-FC	Mesenchymal stem cell	Contralateral	0	Symptomatic
Altanerova, V	2011	Rat	None	C6	Cdy::UPRT	5-FC	Mesenchymal stem cell	Contralateral	0	Symptomatic
Altanerova, V	2011	Rat	None	C6	Cdy::UPRT	5-FC	Mesenchymal stem cell	Contralateral	0	Symptomatic
Altanerova, V	2011	Rat	None	C6	Cdy::UPRT	5-FC	Mesenchymal stem cell	Contralateral	0	Symptomatic
Amano, T	2007	Mouse	None	KR158B	RHAMM mRNA	None	Dendritic cells	Intraperitoneal	>0	Survival
Ambar, B	1999	Rat	None	F98	FasL	None	Adenovirus	Intratumoural	>0	Symptomatic
Andreansky, S	1998	Mouse	None	GL261	IL-10	None	Adenovirus	Intratumoural	>0	Symptomatic
Andreansky, S	1998	Mouse	None	GL261	IL-4	None	Adenovirus	Intratumoural	>0	Symptomatic
Andreansky, S	1998	Mouse	None	GL261	IL-4	None	Adenovirus	Intratumoural	>0	Symptomatic
Andreansky, S	1998	Mouse	None	GL261	IL-4	None	Adenovirus	Intratumoural	>0	Symptomatic
Badie, B	1998	Rat	None	9L	p53	None	Adenovirus	Intratumoural	>0	Survival
Benedetti, S	1997	Rat	None	C6	IL-4	None	Retrovirus	Coinoculation	0	Survival
Benedetti, S	2000	Rat	None	C6	IL-4	None	Neural stem cell	Coinoculation	>0	Unknown
Benedetti, S	2000	Rat	None	C6	IL-4	None	Neural stem cell	Coinoculation	0	Unknown
Benedetti, S	2000	Mouse	None	GL261	GALC	None	Neural stem cell	Coinoculation	0	Unknown

Name	***Year***	***Anima l***	***Comorbidity***	***Glioma Model***	***Gene therapy***	***Prodrug***	***Vector***	***Route of delivery***	***Delay to treatment***	***Survival endpoint***
Benedetti, S	2000	Mouse	None	GL261	GALC	None	Neural stem cell	Coinoculation	>0	Unknown
Benedetti, S	2000	Mouse	None	GL261	IL-4	None	Neural stem cell	Coinoculation	>0	Unknown
Benedetti, S	2000	Mouse	None	GL261	IL-4	None	Neural stem cell	Coinoculation	0	Unknown
Benedetti, S	2003	Rat	None	9L	IL-4	None	Retrovirus-producing cells	Coinoculation	0	Unknown
Benedetti, S	2003	Rat	None	9L	IL-4	None	Retrovirus-producing cells	Coinoculation	0	Unknown
Benedetti, S	2003	Rat	None	9L	IL-4	None	Retrovirus-producing cells	Coinoculation	0	Unknown
Berenstein, M	1998	Rat	None	C6	HSV-TK	GCV	Retrovirus	Intratumoural	<0	Unknown
Berenstein, M	1998	Rat	None	C6	HSV-TK	GCV	Retrovirus	Coinoculation	0	Unknown
Bourbeau, D	2007	Mouse	None	GL261	CU	5-FC	Adenovirus	Intratumoural	>0	Symptomatic
Bourbeau, D	2007	Mouse	None	GL261	CU	5-FC	Adenovirus	Intratumoural	>0	Symptomatic
Bourbeau, D	2007	Mouse	None	GL261	CU	5-FC	Adenovirus	Intratumoural	>0	Symptomatic
Bourbeau, D	2007	Mouse	None	GL261	CU	None	Adenovirus	Intratumoural	>0	Symptomatic
Bourbeau, D	2007	Mouse	Athymic	U87	CU	5-FC	Adenovirus	Intratumoural	>0	Symptomatic
Bourbeau, D	2007	Mouse	Athymic	U87	CU	5-FC	Adenovirus	Intratumoural	>0	Symptomatic
Bourbeau, D	2007	Mouse	Athymic	U87	CU	5-FC	Adenovirus	Intratumoural	>0	Symptomatic
Bourbeau, D	2007	Mouse	Athymic	U87	CU	None	Adenovirus	Intratumoural	>0	Symptomatic
Boviatsis, E	1994	Rat	None	9L	hrR3 (TK)	None	Adenovirus	Intratumoural	>0	Unknown

Name	***Year***	***Anima l***	***Comorbidity***	***Glioma Model***	***Gene therapy***	***Prodrug***	***Vector***	***Route of delivery***	***Delay to treatment***	***Survival endpoint***
Boviatsis, E	1994	Rat	None	9L	RR and TK	None	Adenovirus	Intracerebral	>0	Unknown
Boviatsis, E	1994	Rat	None	9L	RR and TK	None	Adenovirus	Intracerebral	>0	Unknown
Bowers, G	2003	Rat	None	9L	p53	None	Adenovirus	Intratumoural	>0	Symptomatic
Bowers, G	2003	Rat	None	RG2	p53	None	Adenovirus	Intratumoural	>0	Symptomatic
Broaddus, W	1999	Rat	None	RG2	p53	None	Adenovirus	Pretransfected	0	Unknown
Candolfi, M	2010	Rat	None	CNS-1	FasL	None	Adenovirus	Intratumoural	>0	Symptomatic
Candolfi, M	2010	Rat	None	CNS-1	FasL	None	Adenovirus	Intratumoural	>0	Symptomatic
Candolfi, M	2010	Rat	None	CNS-1	TNFα	None	Adenovirus	Intratumoural	>0	Symptomatic
Candolfi, M	2010	Rat	None	CNS-1	TRAIL	None	Adenovirus	Intratumoural	>0	Symptomatic
Candolfi, M	2010	Mouse	Athymic	GBM12	IL-13	None	Adenovirus	Intratumoural	>0	Symptomatic
Candolfi, M	2010	Mouse	None	GL26	IL-13	None	Adenovirus	Intratumoural	>0	Symptomatic
Candolfi, M	2010	Mouse	Athymic	U251	IL-13	None	Adenovirus	Intratumoural	>0	Symptomatic
Candolfi, M	2010	Mouse	Athymic	U251	IL-13	None	Adenovirus	Intratumoural	>0	Symptomatic

Name	Year	Anima l	Comorbidity	Glioma Model	Gene therapy	Prodrug	Vector	Route of delivery	Delay to treatment	Survival endpoint
Candolfi, M	2010	Mouse	Athymic	U251	IL-13	None	Adenovirus	Intratumoural	>0	Symptomatic
Candolfi, M	2010	Mouse	Athymic	U251	IL-13	None	Adenovirus	Intratumoural	>0	Symptomatic
Chambers , R	1995	Mouse	SCID	MT 539	gamma1 34.5	None	Adenovirus	Intratumoural	0	Symptomatic + 1 day
Chambers , R	1995	Mouse	SCID	MT 539	gamma1 34.5	None	Adenovirus	Intratumoural	>0	Symptomatic + 1 day
Chambers , R	1995	Mouse	SCID	MT 539	gamma1 34.5	None	Adenovirus	Intratumoural	0	Symptomatic + 1 day
Chambers , R	1995	Mouse	SCID	MT 539	gamma1 34.5	None	Adenovirus	Intratumoural	0	Symptomatic + 1 day
Chen, J	2006	Mouse	Athymic	C6	sFLK-1	None	Adenovirus	Intravenous	>0	Unknown
Choi, Y	2011	Rat	None	F98	TRAIL	None	Mesenchymal stem cell	Intratumoural	>0	Survival
Ciesielski, M	2006	Mouse	None	GL261	Survivin	None	Plasmids	Subcutaneous	<0	Symptomatic + 1 day
Ciesielski, M	2006	Mouse	None	GL261	Survivin	None	Plasmids	Subcutaneous	<0	Symptomatic + 1 day
Ciesielski, M	2006	Mouse	None	GL261	Survivin	None	Plasmids	Subcutaneous	<0	Symptomatic + 1 day
Cirielli, C	1999	Mouse	Athymic	U251	p53	None	Adenovirus	Pretransfected	0	Survival
Conrad, C	2005	Mouse	Athymic	U87	Delta 24-CD	5-FC	Adenovirus	Intratumoural	>0	Survival
Conrad, C	2005	Mouse	Athymic	U87	Delta 24-CD	5-FC	Adenovirus	Intratumoural	>0	Survival
Conrad, C	2005	Mouse	Athymic	U87	Delta 24-CD	5-FC	Adenovirus	Intratumoural	>0	Survival
Conrad, C	2005	Mouse	Athymic	U87	Delta 24-CD	5-FC	Adenovirus	Intratumoural	>0	Survival
Cool, V	1996	Rat	None	9L	HSV-TK	GCV	Adenovirus	Intratumoural	0	Unknown

Name	Year	Animal	Comorbidity	Glioma Model	Gene therapy	Prodrug	Vector	Route of delivery	Delay to treatment	Survival endpoint
Curtin, J	2009	Mouse	None	GL261	HSV-Flt3L	None	Adenovirus	Intratumoural	>0	Symptomatic
Curtin, J	2009	Mouse	None	GL261	HSV-Flt3L	None	Adenovirus	Intratumoural	>0	Symptomatic
Curtin, J	2009	Mouse	None	GL261	HSV-TK	None	Adenovirus	Intratumoural	>0	Symptomatic
Denbo, J	2011	mouse	SCID	MT330	IFNβ	None	Adenovirus	Intratumoural	>0	Symptomatic
DiMeco, F	2000	Rat	None	9L	IL-12	None	Retrovirus	Pretransfected	>0	Survival
Dmitrieva, N	2011	Mouse	Athymic	U87	Chase	None	Adenovirus	Intracerebral	>0	Symptomatic
Dong, Y	1996	Rat	None	9L	CD	5-FC	Adenovirus	Intratumoural	>0	Survival
Dong, Y	1996	Rat	None	9L	CD	5-FC	Adenovirus	Intratumoural	>0	Survival
Ehtesham, M	2002	Mouse	None	GL261	IFNγ	None	Adenovirus	Intratumoural	>0	Unknown
Ehtesham, M	2002	Mouse	None	GL261	TNFα	None	Adenovirus	Intratumoural	>0	Unknown
Frankel, B	2001	Rat	None	36B10	FasL	None	Plasmids	Pretransfected	0	Symptomatic
Friese, M	2003	Mouse	Athymic	SMA-560	MICA	None	Adenovirus	Subcutaneous	>0	Symptomatic
Fueyo, J	2003	Mouse	Athymic	U87	Rb	None	Adenovirus	Intratumoural	>0	Symptomatic
Galipeau, J	1999	Rat	None	C6	HSV-TK	GCV	Retrovirus	Intratumoural	>0	Unknown
Girald, W	2011	Rat	None	U87	lin/GO	None	Plasmids	Subcutaneous	0	Survival
Girald, W	2011	Rat	None	U87	lin/GO	None	Plasmids	Intratumoural	0	Survival
Glick, R	1997	Mouse	None	GL261	IL-2	None	Fibroblast	Subcutaneous	0	Unknown
Glick, R	1997	Mouse	None	GL261	IL-2	None	Fibroblast	Subcutaneous	0	Unknown
Glick, R	1997	Mouse	None	GL261	IL-2	None	Fibroblast	Subcutaneous	0	Unknown
Glick, R	1997	Mouse	None	GL261	IL-2	None	Fibroblast	Intracerebral	0	Unknown
Glick, R	1997	Mouse	None	GL261	IL-2	None	Fibroblast	Intracerebral	0	Unknown

Name	***Year***	***Anima l***	***Comorbidity***	***Glioma Model***	***Gene therapy***	***Prodrug***	***Vector***	***Route of delivery***	***Delay to treatment***	***Survival endpoint***
Glick, R	1999	Mouse	None	GL261	IL-2	None	Fibroblast	Coinoculation	0	Survival
Glick, R	1999	Mouse	None	GL261	IL-2	None	Fibroblast	Coinoculation	0	Survival
Glick, R	2006	Mouse	None	GL261	IL-2	None	Fibroblast	Coinoculation	0	Unknown
Glick, R	2006	Mouse	None	GL261	IL-2	None	Fibroblast	pretreatment/C oinoculation	<0	Unknown
Glick, R	2006	Mouse	None	GL261	IL-2	None	Fibroblast	Coinoculation	0	Unknown
Goldman, C	1998	mouse	SCID	D54	sFlt-1	None	Plasmids	Pretransfected	0	Unknown
Gomez-Manzanc, C	2004	Mouse	Athymic	D54	E1a and E1b	None	Adenovirus	Intratumoural	>0	Symptomatic
Hamed, H	2010	Mouse	Athymic	GBM14	mda/IL-24	None	Adenovirus	Intratumoural	>0	Symptomatic
Harada, K	1995	Rat	Athymic	T9	IFNβ	None	Lipo/polyplexes	Coinoculation	0	Survival
Harding, T	2006	Mouse	None	4C8	VEGF	None	rAdeno-associated virus	Intratumoural	>0	Symptomatic
Harding, T	2006	Mouse	None	4C8	VEGF	None	rAdeno-associated virus	Intratumoural	>0	Symptomatic
Harding, T	2006	Mouse	None	4C8	VEGF	None	rAdeno-associated virus	Intratumoural	>0	Symptomatic
Harding, T	2006	Mouse	None	C6	VEGF	None	rAdeno-associated virus	Intratumoural	>0	Symptomatic
Harding, T	2006	Mouse	None	C6	VEGF	None	rAdeno-associated virus	Intratumoural	>0	Symptomatic
Harding, T	2006	Mouse	None	C6	VEGF	None	rAdeno-associated virus	Intratumoural	>0	Symptomatic
Harding, T	2006	Mouse	None	U251	VEGF	None	rAdeno-associated virus	Intratumoural	>0	Symptomatic
Harding, T	2006	Mouse	None	U251	VEGF	None	rAdeno-associated virus	Intratumoural	>0	Symptomatic

Name	*Year*	*Animal*	*Comorbidity*	*Glioma Model*	*Gene therapy*	*Prodrug*	*Vector*	*Route of delivery*	*Delay to treatment*	*Survival endpoint*
Harding, T	2006	Mouse	None	U251	VEGF	None	rAdeno-associated virus	Intratumoural	>0	Symptomatic
Harding, T	2006	Rat	Athymic	U251	VEGF	None	rAdeno-associated virus	Intratumoural	>0	Symptomatic
Harding, T	2006	Mouse	None	U87	VEGF	None	rAdeno-associated virus	Intratumoural	>0	Symptomatic
Harding, T	2006	Mouse	None	U87	VEGF	None	rAdeno-associated virus	Intratumoural	>0	Symptomatic
Harding, T	2006	Mouse	None	U87	VEGF	None	rAdeno-associated virus	Intratumoural	>0	Symptomatic
Hasegawa, H	2010	Rat	None	9L	IFNβ	None	Retrovirus	Intratumoural	>0	Symptomatic
Hellums, E	2005	Mouse	None	4C8	HSV-TK and IL-12	None	Adenovirus	Intratumoural	>0	Symptomatic
Hellums, E	2005	Mouse	None	4C8	HSV-TK and IL-12	None	Adenovirus	Intratumoural	>0	Symptomatic
Herrlinger, U	1997	Mouse	None	GL261	GM-CSF	None	Retrovirus	Subcutaneous	<0	Symptomatic
Herrlinger, U	1997	Mouse	None	GL261	GM-CSF	None	Retrovirus	Subcutaneous	>0	Symptomatic
Herrlinger, U	1997	Mouse	None	GL261	GM-CSF	None	Retrovirus	Subcutaneous	>0	Symptomatic
Herrlinger, U	1997	Mouse	None	GL261	GM-CSF	None	Retrovirus	Subcutaneous	<0	Symptomatic
Herrlinger, U	1997	Mouse	None	GL261	GM-CSF	None	Retrovirus	Subcutaneous	<0	Symptomatic
Herrlinger, U	1998	rat	None	D74	HSV-TK	None	Adenovirus	Intratumoural	>0	Symptomatic
Herrlinger, U	1998	rat	None	D74	HSV-TK	None	Adenovirus	Intratumoural	>0	Symptomatic
Hoffman, G	2007	Mouse	Athymic	U251	RGD	None	Adenovirus	Intratumoural	>0	Unknown
Hoffman, G	2007	Mouse	Athymic	U251	RGD	None	Adenovirus	Intratumoural	>0	Unknown
Hoffman, G	2007	Mouse	Athymic	U251	RGD	None	Adenovirus	Intratumoural	>0	Unknown

Name	*Year*	*Anima l*	*Comorbidity*	*Glioma Model*	*Gene therapy*	*Prodrug*	*Vector*	*Route of delivery*	*Delay to treatment*	*Survival endpoint*
Hoffman, G	2007	Mouse	Athymic	U251	RGD	None	Adenovirus	Intratumoural	>0	Unknown
Huang, D	2010	Rat	None	C6	Cx43	GCV	Plasmids	Intratumoural	>0	Survival
Huang, D	2010	Rat	None	C6	HSV-TK	GCV	BMesenchymal stem cell	Intratumoural	>0	Survival
Huang, Q	2007	Rat	None	C6	p53	None	Adenovirus	Intratumoural	>0	Survival
Huang, Q	2010	Rat	None	C6	HSV-TK	ACV	Adenovirus	Intratumoural	>0	Survival
Huang, Q	2010	Rat	None	C6	p53	ACV	Adenovirus	Intratumoural	>0	Survival
Huang, S	2011	Mouse	None	C6	TRAIL	None	Lipo/polyplexes	Intravenous	>0	Unknown
Huang, S	2011	Mouse	None	C6	TRAIL	None	Lipo/polyplexes	Intravenous	>0	Unknown
Huang, S	2011	Mouse	None	C6	TRAIL	None	Lipo/polyplexes	Intravenous	>0	Unknown
Huang, S	2011	Mouse	None	C6	TRAIL	None	Lipo/polyplexes	Intravenous	>0	Unknown
Huszthy, P	2006	Rat	None	BT4C	Human Endostatin	None	Retrovirus	Pretransfected	0	Survival
Huszthy, P	2006	Rat	None	BT4C	Murine Endostatin	None	Retrovirus	Pretransfected	0	Survival
Huszthy, P	2008	Rat	Athymic	GBM1-6	HSV-gamma34.5	None	Adenovirus	Intratumoural	>0	Symptomatic
Huszthy, P	2010	Rat	None	GBM	HSV-TK	GCV	Retrovirus	Intratumoural	>0	Unknown
Huszthy, P	2010	Rat	None	GBM	HSV-TK	GCV	Retrovirus	Intratumoural	>0	Unknown
Ikeda, K	2000	Rat	Athymic	U87	HSV-TK	CPA and CVF	Adenovirus	IArterial	>0	Symptomatic
Ito, S	2010	Mouse	Athymic	U251	CD and IFNβ	5-FC	Neural stem cell	Intravenous	>0	Unknown

Name	Year	Animal	Comorbidity	Glioma Model	Gene therapy	Prodrug	Vector	Route of delivery	Delay to treatment	Survival endpoint
Ito, S	2010	Mouse	None	U251	IFNβ	5-FC	Neural stem cell	Intravenous	>0	Survival
Iwadate, Y	2000	Rat	None	9L	IL-2	None	Retrovirus	Coinoculation	0	Symptomatic
Iwadate, Y	2000	Rat	None	9L	IL-2	None	Retrovirus	Coinoculation	0	Symptomatic
Iwadate, Y	2005	Rat	None	9L irradiated	IL-2	None	Retrovirus	Intratumoural	>0	Survival
Jeong, M	2009	Mouse	Athymic	U87	stTRAIL	None	Adenovirus	Intratumoural	>0	Survival
Jia, Q	2010	Rat	None	C6	Ku70	None	Adenovirus	Pretransfected	>0	Survival
Kanai, R	2006	Mouse	None	U87	dvM345	None	Plasmids	Intratumoural	>0	Survival
Kanai, R	2006	Mouse	None	U87	G207	None	Adenovirus	Pretransfected	>0	Survival
Kanai, R	2011	Mouse	Athymic	BT74	G47-delta	None	Adenovirus	Intratumoural	>0	Symptomatic
Kanai, R	2011	Mouse	Athymic	GBM8	G47-delta	None	Adenovirus	Intratumoural	>0	Symptomatic
Kanai, R	2012	Mouse	Athymic	MGG4	HSV-gamma34.5	None	Adenovirus	Intratumoural	>0	Unknown
Kanai, R	2012	Mouse	Athymic	MGG4	HSV-gamma34.5	None	Adenovirus	Intratumoural	>0	Unknown
Kanai, R	2012	Mouse	None	N18	HSV-gamma34.5	None	Adenovirus	Intratumoural	>0	Unknown
Kanai, R	2012	Mouse	None	N18	HSV-gamma34.5	None	Adenovirus	Intratumoural	>0	Unknown
Kanai, R	2012	Mouse	None	N18	HSV-gamma34.5	None	Adenovirus	Intratumoural	>0	Unknown
Kanai, R	2012	Mouse	Athymic	U87	HSV-gamma34.5	None	Adenovirus	Intratumoural	>0	Unknown
Kanai, R	2012	Mouse	Athymic	U87	HSV-gamma34.5	None	Adenovirus	Intratumoural	>0	Unknown
Kanai, R	2012	Mouse	Athymic	U87	HSV-gamma34.5	None	Adenovirus	Intratumoural	>0	Unknown

Name	Year	Animal	Comorbidity	Glioma Model	Gene therapy	Prodrug	Vector	Route of delivery	Delay to treatment	Survival endpoint
Kato, T	2010	Mouse	SCID	0316-GIC	MGMT siRNA	none	Lipo/polyplexes	Coinoculation	0	Unknown
Kato, T	2010	Mouse	SCID	0316-GIC	MGMT siRNA	TMZ	Lipo/polyplexes	Coinoculation	0	Unknown
Kikuchi, T	1999	Mouse	None	SR-B10.A	IL-2	None	Lipo/polyplexes	Intraperitoneal	>0	Unknown
Kim, C	2006	Mouse	None	GL261	IL-12	None	Dendritic cells	Subcutaneous	<0	Survival
Kim, C	2007	Mouse	None	GL261	TAT-Survivin	None	Dendritic cells	Subcutaneous	>0	Survival
Kim, C	2007	Mouse	None	GL261	TAT-Survivin	TMZ	Dendritic cells	Subcutaneous	>0	Survival
Kim, D	2008	Mouse	Athymic	U87	TRAIL	None	Mesenchymal stem cell	Contralateral	>0	Unknown
Kim, S	2008	Mouse	Athymic	U87	TRAIL	None	Adenovirus	Contralateral	>0	Unknown
Kurozumi, K	2007	Rat	None	D74/HveC	IFNα	None	Adenovirus	Intratumoural	>0	Symptomatic
Kuwashima, N	2005	Mouse	None	GL261	IFNα	None	Dendritic cells	Intratumoural	>0	Symptomatic
Lal, B	2005	Mouse	SCID	U87	c-met and SF-HGF	None	Oligopeptides	Intratumoural	>0	Survival
Lamfers, M	2005	Mouse	None	U87	TIMP-3	None	Adenovirus	Subcutaneous	0	Survival
Lau, C	2009	Mouse	Athymic	U251	FOXO1	None	Adenovirus	Intratumoural	>0	Survival
Lau, C	2009	Mouse	Athymic	U87	FOXO1	None	Adenovirus	Intratumoural	>0	Survival
Lee, E	2011	Mouse	None	U87	HSV-TK	GCV	Neural stem cell	Contralateral	>0	Unknown
Lee, S	2012	Mouse	None	U87	HSV-TK	None	Adenovirus	Intracerebral	>0	Unknown
Lee, S	2012	Mouse	None	U87	HSV-TK	None	Adenovirus	Intracerebral	>0	Unknown
Lee, S	2012	Mouse	None	U87	miR145	None	Adenovirus	Intracerebral	>0	Unknown
Li, C	2007	Rat	None	C6	IL-18	None	BMesenchymal stem cell	Contralateral	>0	Unknown
Li, H	1999	Mouse	Athymic	U87	p53	None	Adenovirus	Intratumoural	>0	Survival

Name	Year	Anima l	Comorbidity	Glioma Model	Gene therapy	Prodrug	Vector	Route of delivery	Delay to treatment	Survival endpoint
Li, H	1999	Mouse	Athymic	U87	p53	None	Adenovirus	Intratumoural	>0	Survival
Li, H	1999	Mouse	Athymic	U87	p53	None	Adenovirus	Intratumoural	>0	Survival
Li, J	2011	Mouse	None	U87	TRAIL	None	Lipo/polyplexes	Intravenous	>0	Survival
Li, J	2011	Mouse	None	U87	TRAIL	None	Lipo/polyplexes	Intravenous	>0	Survival
Li, S	2005	Mouse	None	C6	HSV-TK	GCV	Neural stem cell	Coinoculation	0	Survival
Li, Y	2005	Rat	None	C6	HSV-TK	None	Retrovirus	Coinoculation	0	Survival
Li, Y	2005	Mouse	None	C6	HSV-TK	None	Neural stem cell	Coinoculation	0	Survival
Liang, B	2009	Rat	None	C6	TRAIL	None	Plasmids	Intraperitonealsi lateral	>0	Survival
Liang, C	2009	Rat	None	C6	CD/TRAIL	None	Plasmids	Intraperitonealsi lateral	>0	Survival
Liau, L	1998	Rat	None	C6	TGF-β2	None	Plasmids	Subcutaneous	>0	Survival
Lichtor, T	1995	Mouse	None	GL261	IFNγ	None	Fibroblast	Coinoculation	0	Survival
Lichtor, T	1995	Mouse	None	GL261	IL-2	None	Fibroblast	Coinoculation	0	Survival
Lichtor, T	2002	Mouse	None	GL261	IL-2/Kb	None	Fibroblast	Coinoculation	>0	Unknown
Lichtor, T	2002	Mouse	None	GL261	IL-2/Kb	None	Fibroblast	Coinoculation	<0	Unknown
Lichtor, T	2003	Mouse	None	GL261	IFNγ	None	Fibroblast	Coinoculation	0	Symptomatic
Lichtor, T	2003	Mouse	None	GL261	IL-2	None	Fibroblast	Coinoculation	0	Symptomatic
Lichtor, T	2003	Mouse	None	GL261	IL-2+IFNγ	None	Fibroblast	Coinoculation	0	Symptomatic
Lichtor, T	2003	Mouse	None	GL261	IL-2K	None	Fibroblast	Coinoculation	>0	Symptomatic
Liu, S	2012	Mouse	None	U87	TRAIL	Doxorubic in	Lipo/polyplexes	Intravenous	>0	Survival
Liu, S	2012	Mouse	None	U87	TRAIL	None	Lipo/polyplexes	Intravenous	>0	Survival

Name	Year	Animal	Comorbidity	Glioma Model	Gene therapy	Prodrug	Vector	Route of delivery	Delay to treatment	Survival endpoint
Liu, Y	2002	Mouse	None	GL261	IL-12	None	Adenovirus	Intratumoural	>0	Symptomatic
Liu, Y	2011	Mouse	None	U251	TRAIL	None	Adenovirus	Intratumoural	>0	Symptomatic
Lu, W	2006	Mouse	None	C6	pORF-hTRAIL	None	Plasmids	Intravenous	>0	Symptomatic
Lumniczky, K	2002	Mouse	None	GL261	GM CSF and IL4	None	Adenovirus	Subcutaneous	0	Symptomatic
Lumniczky, K	2002	Mouse	None	GL261	GM-CSF	None	Adenovirus	Subcutaneous	0	Symptomatic
Lumniczky, K	2002	Mouse	None	GL261	GM-CSF	None	Adenovirus	Subcutaneous	0	Symptomatic
Lumniczky, K	2002	Mouse	None	GL261	GM-CSF	None	Adenovirus	Subcutaneous	0	Symptomatic
Lumniczky, K	2002	Mouse	None	GL261	GM-CSF	None	Adenovirus	Subcutaneous	0	Symptomatic
Lumniczky, K	2002	Mouse	None	GL261	GM-CSF	None	Adenovirus	Subcutaneous	0	Symptomatic
Lumniczky, K	2002	Mouse	None	GL261	IL-12	None	Adenovirus	Subcutaneous	0	Symptomatic
Lumniczky, K	2002	Mouse	None	GL261	IL-4	None	Adenovirus	Subcutaneous	0	Symptomatic
Lumniczky, K	2002	Mouse	None	GL261	IL-4	None	Adenovirus	Subcutaneous	0	Symptomatic
Lumniczky, K	2002	Mouse	None	GL261	IL-4	None	Adenovirus	Subcutaneous	0	Symptomatic
Lumniczky, K	2002	Mouse	None	GL261	IL-4	None	Adenovirus	Subcutaneous	0	Symptomatic
Lumniczky, K	2002	Mouse	None	GL261	IL-4	None	Adenovirus	Subcutaneous	0	Symptomatic
Lun, X	2006	Mouse	Athymic	U87	M protein	None	Adeno-associated virus	Intravenous	>0	Symptomatic
Lun, X	2009	Rat	None	F98	EGFP	None	Adenovirus	Intravenous	>0	Symptomatic
Lun, X	2009	Rat	None	RG2	EGFP	None	Adenovirus	Intravenous	>0	Symptomatic
Lun, X	2009	Rat	None	RG2	EGFP	None	Adenovirus	Intravenous	>0	Symptomatic

Name	Year	Animal	Comorbidity	Glioma Model	Gene therapy	Prodrug	Vector	Route of delivery	Delay to treatment	Survival endpoint
Lun, X	2009	Rat	None	RG2	EGFP	None	Adenovirus	Intravenous	>0	Symptomatic
Lun, X	2009	Rat	None	RG2	EGFP	None	Adenovirus	Intravenous	>0	Symptomatic
Ma, H	2002	Rat	None	C6	Angiostatin	None	Adeno-associated virus	Intratumoural	>0	Unknown
Machein, M	1999	Rat	None	GS-9L	VEGFR-2	None	Retrovirus	Intratumoural	0	Symptomatic
Machein, M	1999	Rat	None	GS-9L	VEGFR-2	None	Retrovirus	Intratumoural	0	Symptomatic
Machein, M	1999	Rat	None	GS-9L	VEGFR-2	None	Retrovirus	Intratumoural	0	Symptomatic
Machein, M	1999	Rat	None	GS-9L	VEGFR-2	None	Retrovirus	Intratumoural	0	Symptomatic
Machein, M	1999	Rat	None	GS-9L	VEGFR-2	None	Retrovirus	Intratumoural	0	Symptomatic
Maeda, M	2006	Rat	None	C6	Cre-loxP CALG Tk GFAP	GCV	Adenovirus	Intratumoural	>0	Survival
Maeda, M	2006	Rat	None	C6	Cre-loxP CALN GFAP	GCV	Adenovirus	Intratumoural	>0	Survival
Maguire, C	2008	Mouse	Athymic	U87	IFNβ	None	Adeno-associated virus	Intratumoural	<0	Symptomatic
Maguire, C	2008	Mouse	Athymic	U87	IFNβ	None	Adeno-associated virus	Intratumoural	<0	Symptomatic
Manome, Y	1996	rat	None	9L	Cytochrome P450 2B1	CPA	Retrovirus-producing cells	Intratumoural	>0	Survival
Manome, Y	1996	rat	None	9L	Cytochrome P450 2B1	CPA	Adenovirus	Intratumoural	>0	Survival
Manome, Y	1996	rat	None	9L	Cytochrome P450 2B1	CPA	Adenovirus	Intratumoural	>0	Survival
Manome, Y	1996	rat	None	9L	Cytochrome P450 2B1	CPA	Retrovirus-producing cells	Intratumoural	>0	Survival
Marconi, P	2000	mouse	Athymic	U87	HSV-TK	GCV	Adenovirus	Intratumoural	>0	Unknown
Marconi, P	2000	mouse	Athymic	U87	HSV-TK	GCV	Adenovirus	Intratumoural	>0	Unknown

Name	Year	Anima l	Comorbidity	Glioma Model	Gene therapy	Prodrug	Vector	Route of delivery	Delay to treatment	Survival endpoint
Marconi, P	2000	mouse	Athymic	U87	HSV-TK	None	Adenovirus	Intratumoural	>0	Unknown
Markert, J	2012	Mouse	None	4C8	IL-12	None	Adenovirus	Intratumoural	>0	Symptomatic
Markert, J	2012	Mouse	None	4C8	IL-4	None	Adenovirus	Intratumoural	>0	Symptomatic
Markert, J	2012	Mouse	SCID	D54	IL-12	None	Adenovirus	Intratumoural	>0	Symptomatic
Markert, J	2012	Mouse	SCID	D54MG	IL-12	None	Adenovirus	Intratumoural	>0	Symptomatic
Markert, J	2012	Mouse	SCID	D54MG	IL-4	None	Adenovirus	Intratumoural	>0	Symptomatic
Markert, J	2012	Mouse	SCID	D54MG	IL-4	None	Adenovirus	Intratumoural	>0	Symptomatic
Maron, A	1996	Rat	None	C6	HSV-TK	GCV	Adenovirus	Intratumoural	>0	Unknown
Matsuda, M	2009	Mouse	SCID	U-118	Eg5 siRNA	None	Retrovirus	Intratumoural	>0	Unknown
Matsuda, M	2011	Mouse	None	RSV-M	IL-2	None	Retrovirus	Intratumoural	>0	Survival
Matsuda, M	2011	Mouse	None	RSV-M	IL-2	None	Mesenchymal stem cell	Intratumoural	>0	Survival
Matsuda, M	2011	Mouse	None	RSV-M	IL-2	None	Retrovirus	Intratumoural	>0	Survival
Meijer, D	2009	Mouse	Athymic	U87	IFNβ	None	Adeno-associated virus	Intraperitonealsi lateral	>0	Symptomatic
Meijer, D	2009	Mouse	Athymic	U87	IFNβ	None	Adeno-associated virus	Intraperitonealsi lateral	>0	Symptomatic
Meijer, D	2009	Mouse	Athymic	U87	IFNβ	None	Adeno-associated virus	Intraperitonealsi lateral	<0	Symptomatic
Mineta, T	1995	Mouse	Athymic	U87	ICP6	None	Adenovirus	Intratumoural	>0	Unknown
Miura, F	2002	Rat	None	9L	HSV-TK	GCV	Adenovirus	Intratumoural	>0	Symptomatic

Name	***Year***	***Anima l***	***Comorbidity***	***Glioma Model***	***Gene therapy***	***Prodrug***	***Vector***	***Route of delivery***	***Delay to treatment***	***Survival endpoint***
Miura, F	2002	Rat	None	9L	HSV-TK	GCV	Adenovirus	Intratumoural	>0	Symptomatic
Mori, K	2010	Rat	None	9L	HSV-TK	GCV	BMesenchymal stem cell	Coinoculation	0	Unknown
Mori, K	2010	Rat	None	9L	HSV-TK	GCV	Mesenchymal stem cell	Coinoculation	0	Survival
Morioka, M	2002	Mouse	None	RSV-M	B7.1	None	Adenovirus	Coinoculation	>0	Survival
Morioka, M	2002	Mouse	None	RSV-M	B7.1	None	Adenovirus	Coinoculation	>0	Survival
Moriuchi, S	1998	mouse	Athymic	U87	TNFα	GCV	Adenovirus	Intratumoural	>0	Unknown
Moriuchi, S	1998	mouse	Athymic	U87	TNFα	GCV	Adenovirus	Intratumoural	>0	Unknown
Moriuchi, S	2000	Rat	None	9L	ICP22 and ICP27	None	Adenovirus	Intratumoural	>0	Symptomatic
Moriuchi, S	2000	Rat	None	9L	ICP4	None	Adenovirus	Intratumoural	>0	Symptomatic
Moriuchi, S	2005	Mouse	Athymic	U87	HSV-TK	GCV	Adenovirus	Intratumoural	>0	Survival
Moriuchi, S	2005	Mouse	Athymic	U87	HSV-TK/IκBαM	None	Adenovirus	Intratumoural	>0	Survival
Nafe, C	2003	Rat	None	9L	HSV-TK	GCV	Virus-producing cells	Intratumoural	>0	Unknown
Nam, M	1996	Rat	None	9L	IL-2	None	EndotheLipo/polyplexesal cells	Coinoculation	0	Survival
Namba, H	1998	Rat	None	9L	HSV-TK	None	Retrovirus	Intratumoural	>0	Survival
Namba, H	1998	Rat	None	9L	HSV-TK	None	Retrovirus	Intratumoural	>0	Survival
Natsume, A	1999	Mouse	None	GL261	IFNβ	None	Lipo/polyplexes	Intratumoural	>0	Survival
Nestler, U	2004	Mouse	Athymic	U87	HSV-TK	None	Adenovirus	Intratumoural	>0	Unknown

Name	***Year***	***Anima l***	***Comorbidity***	***Glioma Model***	***Gene therapy***	***Prodrug***	***Vector***	***Route of delivery***	***Delay to treatment***	***Survival endpoint***
Niranjan, A	2000	Mouse	Athymic	U87	HSV-TK	None	Adenovirus	Intratumoural	>0	Symptomatic
Niranjan, A	2000	mouse	Athymic	U87	TNFα	None	Adenovirus	Intratumoural	>0	Symptomatic
Ogren, S	2009	Rat	Athymic	U87	DTEGF13	None	Plasmids	Intratumoural	>0	Unknown
Ohlfest, J	2005	Mouse	None	U87	sFlt-1 and statin-AE	None	Plasmids	Intratumoural	>0	Survival
Ohlfest, J	2005	Mouse	None	U87	sFlt-1 and statin-AE	None	Plasmids	Intratumoural	>0	Survival
Okada, T	2001	Rat	None	9L	HSV-TK	GCV	Adenovirus	Subcutaneous	>0	Unknown
Otsuki, A	2008	Mouse	None	U87	Nestin	None	Adenovirus	Intratumoural	>0	Unknown
Otsuki, A	2008	Mouse	None	U87	Nestin	Valproic acid	Adenovirus	Intratumoural	>0	Unknown
Paraskeva kou, G	2007	Mouse	None	GBM12	EGFR	None	Retrovirus	Intratumoural	>0	Survival
Paraskeva kou, G	2007	Mouse	None	GBM12	virus only	None	Retrovirus	Intratumoural	>0	Survival
Paul, D	2000	Rat	None	F98	B7.1	None	Plasmids	Subcutaneous	<0	Symptomatic + 1 day
Paul, S	2000	Rat	None	F98	B7.1	None	Plasmids	Subcutaneous	<0	Symptomatic + 1 day
Pellegatta, S	2006	Mouse	None	GL261-AC	GL261-AC	None	Dendritic cells	Subcutaneous	>0	Survival
Pellegatta, S	2006	Mouse	None	GL261-NS	GL261-AC	None	Dendritic cells	Subcutaneous	>0	Survival
Pellegatta, S	2006	Mouse	None	GL261-AC	GL261-NS	None	Dendritic cells	Subcutaneous	>0	Survival
Pellegatta, S	2006	Mouse	None	GL261-NS	GL261-NS	None	Dendritic cells	Subcutaneous	>0	Survival
Peres, E	2011	Rat	None	9L	EPO	None	Plasmids	Pretransfected	0	Unknown
Peres, E	2011	Rat	None	9L	EPO	None	Plasmids	Pretransfected	0	Unknown
Peres, E	2011	Mouse	None	U87	EPO	None	Plasmids	Pretransfected	0	Unknown

Name	*Year*	*Anima l*	*Comorbidity*	*Glioma Model*	*Gene therapy*	*Prodrug*	*Vector*	*Route of delivery*	*Delay to treatment*	*Survival endpoint*
Peres, E	2011	Mouse	None	U87	EPO-R	None	Plasmids	Pretransfected	0	Unknown
Perri, S	2005	Mouse	None	U87	U87-hk5His-GFP	None	Retrovirus	Pretransfected	0	Survival
Persson, B	2010	Rat	None	N29	IFNγ	None	Retrovirus	Intraperitoneal	>0	Symptomatic
Persson, B	2010	Rat	None	N32	IFNγ	None	Retrovirus	Intraperitoneal	>0	Symptomatic
Phuong, L	2003	Mouse	Athymic	U87	CEA Mv	None	Retrovirus	Intratumoural	>0	Symptomatic
Redaelli, M	2012	Rat	None	F98	HSV-TK	GCV	Adenovirus	Intravenous	>0	Survival
Redaelli, M	2012	Mouse	None	GL261	HSV-TK	GCV	Adenovirus	Intravenous	>0	Survival
Roche, F	2010	Rat	None	RG2	IL-12 and VLP	None	Retrovirus	Intratumoural	>0	Symptomatic
Ross, J	1995	Rat	None	9L	HSV-TK	GCV	Adenovirus	Intracerebral	>0	Unknown
Ryu, C	2011	Mouse	None	GL26	IL-12	None	Mesenchymal stem cell	Intratumoural	>0	Unknown
Saka, M	2010	Mouse	None	KR158B	IL-13ra2	None	Dendritic cells	Intraperitoneal	>0	Unknown
Samoto, K	2002	Mouse	Athymic	U87	HSV-TK	None	Adenovirus	Intratumoural	>0	Unknown
Sandmair, A	2000	Rat	None	BT4C	HSV-TK	GCV	Retrovirus	Pretransfected	0	Survival
Santra, M	2010	Rat	Athymic	U87	DCX	None	Retrovirus	Intratumoural	>0	Survival
Sato, H	2005	Mouse	None	GL261	IFNα	None	Mesenchymal stem cell	Intratumoural	>0	Survival
Schneider , T	2008	Rat	None	F98	TGF-β	None	Oligopeptides	Subcutaneous	0	Survival
Shah, A	2006	Mouse	SCID	D54	IL-12	None	Adenovirus	Intratumoural	>0	Survival
Shah, A	2006	Mouse	SCID	D54	IL-12	None	Adenovirus	Intratumoural	>0	Survival

Name	***Year***	***Animal***	***Comorbidity***	***Glioma Model***	***Gene therapy***	***Prodrug***	***Vector***	***Route of delivery***	***Delay to treatment***	***Survival endpoint***
Shah, A	2006	Mouse	SCID	D54MG	IL-12	None	Adenovirus	Intratumoural	>0	Survival
Shah, A	2007	Mouse	SCID	U87	HSV-34.5gamma and IRS1	None	Adenovirus	Intratumoural	>0	Symptomatic
Shah, A	2007	Mouse	SCID	U87	HSV-34.5gamma and IRS1	None	Adenovirus	Intratumoural	>0	Symptomatic
Shah, A	2007	Mouse	SCID	U87	HSV-34.5gamma and TRS1	None	Adenovirus	Intratumoural	>0	Symptomatic
Shah, A	2007	Mouse	SCID	U87	HSV-34.5gamma and TRS1	None	Adenovirus	Intratumoural	>0	Symptomatic
Shir, A	2006	Mouse	Athymic	U87	Poly IC	None	Lipo/polyplexes	Intratumoural	>0	Unknown
Sonabend, A	2008	Mouse	None	GL261	IL-12	BCNU	Lipo/polyplexes	Intratumoural	>0	Survival
Sonabend, A	2008	Mouse	None	GL261	IL-12	None	Lipo/polyplexes	Intratumoural	>0	Survival
Sonabend, A	2008	Mouse	None	GL261	IL-12	None	Lipo/polyplexes	Intratumoural	>0	Survival
Sun, X	2012	Mouse	None	U87	TRAIL	None	Lipo/polyplexes	Intravenous	>0	Survival
Sun, X	2012	Mouse	None	U87	TRAIL	None	Lipo/polyplexes	Intravenous	>0	Survival
Sun, X	2012	Mouse	None	U87	TRAIL	Paclitaxel	Lipo/polyplexes	Intravenous	>0	Survival
Sun, X	2012	Mouse	None	U87	TRAIL	Paclitaxel	Lipo/polyplexes	Intravenous	>0	Survival
Szatmari, T	2008	Mouse	None	C6	dCK	Gemcitibine	Adenovirus	Pretransfected	0	Symptomatic
Szatmari, T	2008	Rat	None	C6	dCK	None	Adenovirus	Pretransfected	0	Symptomatic
Szatmari, T	2008	Mouse	None	GL261	dCK	Gemcitibine	Adenovirus	Pretransfected	0	Symptomatic

Name	Year	Anima l	Comorbidity	Glioma Model	Gene therapy	Prodrug	Vector	Route of delivery	Delay to treatment	Survival endpoint
Szatmari, T	2008	Mouse	None	GL261	dCK	None	Adenovirus	Pretransfected	0	Symptomatic
Tabatabai, G	2010	Mouse	None	LNT-229	HPC	None	Retrovirus	Intravenous	>0	Symptomatic
Tabatabai, G	2010	Mouse	None	SMA-560	HPC	None	Retrovirus	Intravenous	>0	Symptomatic
Tamura, K	2001	Mouse	None	RSV-M	HSV-TK	GCV	Retrovirus	Intratumoural	>0	Survival
Tse, V	2004	Rat	None	Rt-2	p53	None	Retrovirus	Pretransfected	0	Symptomatic
Tseng, S	1997	Rat	None	C6	GM-CSF	None	Retrovirus	Intraperitoneal	0	Survival
Tseng, S	1997	Rat	None	C6	IL-2	None	Retrovirus	Intraperitoneal	0	Survival
Tseng, S	1997	Rat	None	C6	IL-4	None	Retrovirus	Intraperitoneal	0	Survival
Tsugawa, T	2004	Mouse	None	GL261	IFNα	None	Adenovirus	Intratumoural	>0	Symptomatic
Ueda, R	2008	Mouse	None	GL261	SOX6	None	Plasmids	Intraperitoneal	>0	Survival
Ueda, R	2008	Mouse	None	GL261	SOX6	None	Plasmids	Intraperitoneal	>0	Survival
Ulasov, I	2007	Mouse	None	GL261	E1A	None	Adenovirus	Intratumoural	>0	Survival
Ulasov, I	2007	Mouse	None	GL261	E1A	None	Adenovirus	Intratumoural	>0	Survival
Ulasov, I	2007	Mouse	Athymic	U87	E1A	None	Adenovirus	Intratumoural	>0	Unknown
Verheije, M	2009	Mouse	Athymic	U87	EGFR	None	Retrovirus	Intratumoural	>0	Symptomatic
Viita, H	2012	Rat	None	BT4C	15-lipoxygenase-1	None	Adenovirus	Intraperitonealsi lateral	>0	Symptomatic
Vincent, A	1996	Rat	None	9L	HSV-TK and IL-2	GCV	Adenovirus	Intratumoural	>0	Unknown

Name	***Year***	***Anima l***	***Comorbidity***	***Glioma Model***	***Gene therapy***	***Prodrug***	***Vector***	***Route of delivery***	***Delay to treatment***	***Survival endpoint***
Vincent, A	1996	Rat	None	9L	HSV-TK and IL-2	GCV	Adenovirus	Intratumoural	>0	Unknown
Vincent, A	1996	Rat	None	9L	HSV-TK and IL-2	GCV	Adenovirus	Intratumoural	>0	Unknown
Vincent, A	1996	Rat	None	9L	HSV-TK and IL-2	GCV	Retrovirus-producing cells	Intratumoural	>0	Unknown
Vincent, A	1996	Rat	None	9L	HSV-TK and IL-2	GCV	Adenovirus	Intratumoural	>0	Unknown
Vincent, A	1996	Rat	None	9L	HSV-TK and IL-2	None	Retrovirus-producing cells	Intratumoural	>0	Unknown
Vincent, A	1997	Rat	None	9L	HSV-TK	GCV	Adenovirus	Intratumoural	>0	Symptomatic
Vincent, A	1997	Rat	None	9L	HSV-TK	GCV	Adenovirus	Intratumoural	>0	Symptomatic
Vincent, A	1997	Rat	None	9L	HSV-TK	GCV	Adenovirus	Intratumoural	>0	Symptomatic
Vincent, A	1997	Rat	None	9L	HSV-TK	GCV	Adenovirus	Intratumoural	>0	Symptomatic
Vincent, A	1997	Rat	None	9L	HSV-TK	GCV	Adenovirus	Intratumoural	>0	Symptomatic
Vincent, A	1997	Rat	None	9L	HSV-TK	GCV	Adenovirus	Intratumoural	>0	Symptomatic
Visse, E	1999	Rat	None	N32	B7.1	None	Retrovirus	Subcutaneous	>0	Symptomatic
Visse, E	1999	Rat	None	N32	B7.1	None	Retrovirus	Subcutaneous	>0	Symptomatic
Visse, E	1999	Rat	None	N32	IFNγ	None	Retrovirus	Subcutaneous	>0	Symptomatic
Visse, E	1999	Rat	None	N32	IFNγ	None	Retrovirus	Subcutaneous	>0	Symptomatic
Visse, E	1999	Rat	None	N32	IL-7	None	Retrovirus	Subcutaneous	>0	Symptomatic

Name	*Year*	*Anima l*	*Comorbidity*	*Glioma Model*	*Gene therapy*	*Prodrug*	*Vector*	*Route of delivery*	*Delay to treatment*	*Survival endpoint*
Visse, E	1999	Rat	None	N32	IL-7	None	Retrovirus	Subcutaneous	>0	Symptomatic
Wang, R	1999	Rat	None	C6	CD and HSV-TK	None	Adenovirus	Intratumoural	>0	Survival
Wang, S	2001	Rat	None	Rt-2	p21	None	Retrovirus	Intraperitonealsi lateral	>0	Survival
Wang, S	2001	Rat	None	Rt-2	p53	None	Retrovirus	Intraperitonealsi lateral	>0	Survival
Wang, T	2001	Rat	None	Rt-2	p16	None	Retrovirus	Intraperitonealsi lateral	>0	Survival
Wang, Y	2011	Mouse	None	U87	siRNA survivin	None	Plasmids	Intravenous	>0	Survival
Wei, M	1995	Mouse	Athymic	C6	IL-4	None	Retrovirus	Intratumoural	>0	Unknown
Wirth, T	2012	Rat	None	BT4C	Avidin	Yttrium	Retrovirus	Intravenous	>0	Survival
Wirth, T	2012	Rat	None	BT4C	Avidin	Yttrium	Retrovirus	Intravenous	>0	Survival
Wu, A	2007	Mouse	None	GL261	IFNγ	None	Plasmids	Intratumoural	>0	Survival
Wu, D	2007	Mouse	None	GL261	IFNγ + SB	None	Plasmids	Intratumoural	>0	Survival
Wu, D	2007	Mouse	None	GL261	IFNγ + SB	None	Plasmids	Intratumoural	>0	Survival
Xu, D	2006	Rat	None	C6	p53	None	Adenovirus	Intracerebral	>0	Survival
Yacoub, A	2003	Rat	None	Rt-2	IL-24	None	Adenovirus	Pretransfected	0	Survival
Yacoub, A	2008	Mouse	Athymic	GBM12	mda-7	None	Adenovirus	Intratumoural	>0	Symptomatic
Yacoub, A	2008	Mouse	Athymic	GBM6	mda-7	None	Adenovirus	Intratumoural	>0	Symptomatic
Yamanak a, R	1995	Mouse	Athymic	NP-1	IL-8	None	Retrovirus	Pretransfected	0	Unknown
Yamanak a, R	1995	Mouse	Athymic	NP-1	IL-8	None	Retrovirus	Pretransfected	0	Unknown
Yamanak a, R	1995	Mouse	Athymic	NP-1	IL-8	None	Retrovirus	Pretransfected	0	Unknown
Yamanak a, R	1999	Mouse	Athymic	Onda 10	TGF-β1	None	Retrovirus	Intracerebral	0	Unknown
Yamanak a, R	1999	Mouse	Athymic	Onda 10	TGF-β1	None	Retrovirus	Intracerebral	0	Unknown

Name	***Year***	***Animal***	***Comorbidity***	***Glioma Model***	***Gene therapy***	***Prodrug***	***Vector***	***Route of delivery***	***Delay to treatment***	***Survival endpoint***
Yamanaka, R	1999	Mouse	Athymic	Onda 10	TGF-β1	None	Retrovirus	Intracerebral	0	Unknown
Yamanaka, R	2000	Mouse	None	G203	IL-12	None	Retrovirus	Pretransfected	>0	Survival
Yamanaka, R	2001	Mouse	None	B16	SFV-mediated B16 cDNA	None	Dendritic cells	Intraperitoneal	<0	Survival
Yamanaka, R	2001	Mouse	None	B16	SFV-mediated G203 cDNA	None	Dendritic cells	Intraperitoneal	<0	Survival
Yamanaka, R	2001	Mouse	None	G203	SFV-mediated B16 cDNA	None	Dendritic cells	Intraperitoneal	<0	Survival
Yamanaka, R	2001	Mouse	None	G203	SFV-mediated G203 cDNA	None	Dendritic cells	Intraperitoneal	<0	Survival
Yamanaka, R	2002	Mouse	None	G203	IL-12 and IL-18	None	Dendritic cells	Intraperitoneal	>0	Unknown
Yamanaka, R	2002	Mouse	None	G203	IL-12 and IL-18	None	Dendritic cells	Intraperitoneal	>0	Unknown
Yamini, B	2004	Mouse	Athymic	U87	TNFα	None	Adenovirus	Intratumoural	>0	Symptomatic
Yamini, B	2004	Mouse	Athymic	U87	TNFα	TMZ	Adenovirus	Intratumoural	>0	Symptomatic
Yang, Y	2011	Mouse	SCID	GBM	sh-STAT3	None	Retrovirus	Unknown	>0	Unknown
Yokoyama, T	2008	Mouse	Athymic	U87	RGD	None	Adenovirus	Intratumoural	>0	Symptomatic
Yokoyama, T	2008	Mouse	Athymic	U87	RGD	RAD001	Adenovirus	Intratumoural	>0	Symptomatic
Yokoyama, T	2008	Mouse	Athymic	U87	RGD	TMZ	Adenovirus	Intratumoural	>0	Symptomatic
Yoo, J	2012	Mouse	None	Gli36delta5	HSV-34.5gamma and Vstat120	None	Adenovirus	Intratumoural	>0	Symptomatic
Yoo, J	2012	Mouse	None	Gli36delta5	HSV-Vstat120	None	Adenovirus	Intratumoural	>0	Symptomatic

Name	***Year***	***Animal***	***Comorbidity***	***Glioma Model***	***Gene therapy***	***Prodrug***	***Vector***	***Route of delivery***	***Delay to treatment***	***Survival endpoint***
Yoo, J	2012	Mouse	None	U87	HSV-34.5gamma and Vstat120	None	Adenovirus	Intratumoural	>0	Symptomatic
Yoo, J	2012	Mouse	None	U87	HSV-gamma34.5	None	Adenovirus	Intratumoural	>0	Symptomatic
Yoo, J	2012	Mouse	None	U87	HSV-Vstat120	None	Adenovirus	Intratumoural	>0	Symptomatic
Yoo, J	2012	Mouse	None	X12-V2	HSV-34.5gamma and Vstat120	None	Adenovirus	Intratumoural	>0	Symptomatic
Yoo, J	2012	Mouse	None	X12-V2	HSV-Vstat120	None	Adenovirus	Intratumoural	>0	Symptomatic
Yoshida, J	2002	Mouse	Athymic	U251	IFNβ	None	Adeno-associated virus	Intratumoural	0	Survival
Yoshida, J	2002	Mouse	Athymic	U251	IFNβ	None	Adeno-associated virus	Intratumoural	0	Survival
Yoshikawa, K	2000	Mouse	None	RSV-MG	IL-4	None	Adenovirus	Pretransfected	>0	Unknown
Yoshikawa, K	2000	Mouse	None	RSV-MG	IL-4	None	Adenovirus	Pretransfected	0	Unknown
Yu, J	1997	Mouse	None	GL261	GM-CSF	None	Retrovirus	Subcutaneous	>0	Survival
Yu, J	1997	Mouse	None	GL261	GM-CSF	None	Retrovirus	Subcutaneous	>0	Survival
Yu, J	1997	Mouse	None	GL261	GM-CSF	None	Retrovirus	Subcutaneous	<0	Survival
Yu, J	1997	Mouse	None	GL261	GM-CSF	None	Retrovirus	Subcutaneous	>0	Survival
Yu, J	1997	Mouse	None	GL261	GM-CSF	None	Retrovirus	Subcutaneous	<0	Survival

Name	***Year***	***Anima l***	***Comorbidity***	***Glioma Model***	***Gene therapy***	***Prodrug***	***Vector***	***Route of delivery***	***Delay to treatment***	***Survival endpoint***
Zang, H	2007	Rat	None	C6	M protein	None	Plasmids	Intravenous	>0	Survival
Zhang, W	2011	Mouse	Athymic	U87	Angiostatin	None	Adenovirus	Intratumoural	>0	Symptomatic
Zhang, W	2011	Mouse	Athymic	U87	Angiostatin	None	Adenovirus	Intratumoural	>0	Symptomatic
Zhang, X	2004	Rat	None	C6	IL-18+Fas	None	Retrovirus	Pretransfected	0	Survival
Zhang, Y	2002	Mouse	SCID	U87	EGFR AS	None	Plasmids	Intravenous	>0	Survival
Zhang, Y	2004	Rat	None	C6	IL-18	None	Retrovirus	Pretransfected	0	Survival
Zhang, Y	2004	Mouse	SCID	U87	EGFR	None	Plasmids	Intravenous	>0	Survival
Zhang, Z	2004	Mouse	Athymic	U87	GALV.fus	None	Plasmids	Intratumoural	>0	Symptomatic
Zhu, F	2011	Mouse	Athymic	U87	sGC-a1b1cys105	None	Retrovirus	Pretransfected	0	Symptomatic
Zhu, H	2011	Mouse	Athymic	U87	sGC	None	Retrovirus	Pretransfected	0	Symptomatic
