# Supplementary material for: A systematic review and meta‐analysis of gene therapy in animal models of cerebral glioma: why did promise not translate to human therapy?
Source: Evid Based Preclin Med. 2015 Jan 20;1(1):e00006. doi: 10.1002/ebm2.6 (PMC5020579; doi:10.1002/ebm2.6)
Supplement: Supplementary file 3 — Appendix S3. Collated gene therapies. [file EBM2-1-21-s001.pdf]

Supplementary material 3: Collated gene therapies

Gene therapy	Number of comparisons	Group
HSV-TK	45	Oncolytic
IL-4	23	Immune Modulation
IL-2	21	Immune Modulation
TRAIL	18	Immune Modulation
GM-CSF	16	Immune Modulation
IL-12	15	Immune Modulation
p53	13	Oncolytic
VEGF	13	Anti-angiogenesis
IFNβ	12	Immune Modulation
HSV-γ34.5	10	Oncolytic
Cdy::UPRT	8	Oncolytic
CU	8	Oncolytic
IFNγ	8	Immune Modulation
RGD	7	Other
TNFα	7	Immune Modulation
B7.1	6	Immune Modulation
HSV-TK and IL-2	6	Multiple
IL-13	6	Immune Modulation
EGFP	5	Oncolytic
VEGFR-2	5	Anti-angiogenesis
Cytochrome P450 2B1	4	Oncolytic
dCK	4	Oncolytic
Delta 24-CD	4	Oncolytic
E1A	4	Oncolytic
FasL	4	Oncolytic
γ1 34.5	4	Oncolytic
IFNα	4	Immune Modulation
Angiostatin	3	Anti-angiogenesis
CD	3	Oncolytic
EGFR	3	Oncolytic
EPO	3	Other
HSV-34.5gamma and Vstat120	3	Multiple
HSV-Vstat120	3	Oncolytic
IL-8	3	Immune Modulation
Survivin	3	Oncolytic
TGF-β1	3	Immune Modulation
Avidin	2	Oncolytic
FOXO1	2	DNA repair
G47-delta	2	Oncolytic
GALC	2	Oncolytic
GL261-AC	2	Other
GL261-NS	2	Other
HPC	2	Other
HSV-34.5γ and IRS1	2	Multiple
HSV-34.5γ and TRS1	2	Multiple
HSV-Flt3L	2	Immune Modulation
HSV-fml-TK	2	Oncolytic
HSV-TK and IL-12	2	Multiple
IFNγ and SB	2	Multiple
IL-12 and IL-18	2	Multiple

Gene therapy	Number of comparisons	Group
IL-18	2	Immune Modulation
IL-2/Kb	2	Multiple
IL-7	2	Immune Modulation
lin/GO	2	Oncolytic
M protein	2	Oncolytic
mda-7	2	Oncolytic
MGMT siRNA	2	Oncolytic
Nestin	2	Oncolytic
RR and TK	2	Multiple
S-pk7	2	Oncolytic
sFlt-1 and statin-AE	2	Multiple
SFV-mediated B16 cDNA	2	Other
SFV-mediated G203 cDNA	2	Other
SOX6	2	Immune Modulation
TAT-Survivin	2	Oncolytic
15-lipoxygenase-1	1	Oncolytic
c-met and SF-HGF	1	Multiple
CD and HSV-TK	1	Multiple
CD and IFNβ	1	Multiple
CD and UPRT	1	Multiple
CD and TRAIL	1	Multiple
CEA Mv	1	Other
Chase	1	Other
Cre-loxP CALG Tk GFAP	1	Oncolytic
Cre-loxP CALN GFAP	1	Other
Cx43	1	Other
DCX	1	DNA repair
DTEGF13	1	Other
dvM345	1	Oncolytic
E1a and E1b	1	Multiple
Eg5 siRNA	1	Oncolytic
EGFR AS	1	Oncolytic
EPO-R	1	Other
G207	1	Oncolytic
GALV.fus	1	Oncolytic
GM CSF and IL4	1	Multiple
HER2	1	Immune Modulation
hrR3 (TK)	1	Oncolytic
HSV-γ34.5 and RR	1	Multiple
HSV-TK and IκBαM	1	Multiple
Human Endostatin	1	Anti-angiogenesis
ICP22 and ICP27	1	Multiple
ICP4	1	Oncolytic
ICP6	1	Oncolytic
IL-10	1	Immune Modulation
IL-12 and VLP	1	Multiple
IL-13ra2	1	Immune Modulation
IL-18 and Fas	1	Multiple
IL-2 and IFNγ	1	Multiple
IL-24	1	Immune Modulation
IL-2K	1	Immune Modulation

Gene therapy	Number of comparisons	Group
Ku70	1	Other
MICA	1	Immune Modulation
miR145	1	Oncolytic
Murine Endostatin	1	Anti-angiogenesis
MV-GFP	1	Oncolytic
MV-GFP/HAA-IL-13	1	Multiple
p16	1	Oncolytic
p21	1	Oncolytic
Poly IC	1	Oncolytic
pORF-hTRAIL	1	Immune Modulation
RAD001	1	Oncolytic
Rb	1	Oncolytic
RHAMM mRNA	1	DNA repair
sFLK-1	1	Other
sFlt-1	1	Anti-angiogenesis
sGC	1	Other
sGC-a1b1cys105	1	Other
sh-STAT3	1	Other
siRNA survivin	1	Oncolytic
stTRAIL	1	Immune Modulation
TGF-β	1	Immune Modulation
TGF-β2	1	Immune Modulation
TIMP-3	1	Other
U87-hk5His-GFP	1	Anti-angiogenesis
Virus only	1	Other
