# Supplementary material for: A systematic review and meta‐analysis of gene therapy in animal models of cerebral glioma: why did promise not translate to human therapy?
Source: Evid Based Preclin Med. 2015 Jan 20;1(1):e00006. doi: 10.1002/ebm2.6 (PMC5020579; doi:10.1002/ebm2.6)
Supplement: Supplementary file 4 — Appendix S4. Study quality scores. [file EBM2-1-21-s002.pdf]

Supplementary Material 4: Study Quality Scores

1) Peer-reviewed publication
2) Standardised number or volume of tumour cells implanted
3) Randomized allocation of tumour-bearing mice into treatment and control groups
4) Blinded assessment of outcome
5) Sample size calculation performed
6) Compliance with animal welfare policy
7) Statement of potential conflict of interests
8) Reported number of animals in which the tumour did not grow
9) Reporting and explanation of excluded animals
10) Evidence of successful transduction and gene expression *in vitro*
11) Evidence of infection, replication and expression *in vivo*

Name	***Year***	***TK model***	***1***	***2***	***3***	***4***	***5***	***6***	***7***	***8***	***9***	***10***	***11***	***Quality Score***
Adachi, Y	2000	No	+	+				+						3
Aghi, M	2006	No	+	+				+				+	+	3
Ahmed, A	2011	No	+	+				+				+		3
Ahmed, N	2010	No	+	+				+				+		3
Ali, S	2005	No	+	+				+				+		3
Allen, C	2008	No	+	+				+		+	+	+	+	5
Alonso, M	2007	No	+	+				+				+	+	3
Alonso, M	2008	No	+	+	+							+		3
Altanerova, V	2011	No	+	+	+			+		+				5
Amano, T	2007	No	+	+					+				+	3
Ambar, B	1999	No	+	+								+		2
Andreansky, S	1998	No	+					+				+		2
Badie, B	1998	No	+	+				+			+			4
Benedetti, S	1997	No	+					+						2
Benedetti, S	2000	No	+	+				+					+	3
Benedetti, S	2003	No	+										+	1
Berenstein, M	1998	Yes	+	+				+						3
Bourbeau, D	2007	No	+	+				+	+			+	+	4
Boviatsis, E	1994	No	+					+					+	2
Boviatsis, E	1994	No	+	+				+					+	3
Bowers, G	2003	No	+	+				+				+		3
Broaddus, W	1999	No	+					+	+			+		3
Candolfi, M	2010	No	+	+				+			+		+	4
Candolfi, M	2010	No	+	+							+	+	+	3
Chambers, R	1995	No	+					+						2
Chen, J	2006	No	+	+							+			3
Choi, Y	2011	No	+	+							+	+	+	3
Ciesielski, M	2006	No	+	+	+			+			+	+		5
Cirielli, C	1999	No	+	+				+				+		3
Conrad, C	2005	No	+	+								+	+	2
Cool, V	1996	Yes	+	+				+						3
Curtin, J	2009	No	+	+						+		+		3
Denbo, J	2011	No	+	+	+					+	+		+	5

Name	*Year*	*TK model*	*1*	*2*	*3*	*4*	*5*	*6*	*7*	*8*	*9*	*10*	*11*	*Quality Score*
DiMeco, F	2000	No	+	+				+				+		3
Dmitrieva, N	2011	No	+	+	+					+	+	+		3
Dong, Y	1996	No	+	+								+	+	3
Ehtesham, M	2002	No	+	+							+		+	3
Frankel, B	2001	No	+	+				+				+		3
Friese, M	2003	No	+	+				+			+	+		5
Fueyo, J	2003	No	+	+	+	+		+				+	+	3
Galipeau, J	1999	Yes	+	+				+		+	+	+	+	3
Girald, W	2011	No	+	+				+				+		5
Glick, R	1997	No	+					+				+		3
Glick, R	1999	No	+	+				+				+		2
Glick, R	2006	No	+	+								+		2
Goldman, C	1998	No	+	+								+		4
Gomez-Manzanc, C	2004	No	+	+				+			+	+	+	2
Hamed, H	2010	No	+	+				+				+	+	3
Harada, K	1995	No	+					+				+		1
Harding, T	2006	No	+									+	+	3
Hasegawa, H	2010	No	+	+				+				+	+	4
Hellums, E	2005	No	+	+				+				+		2
Herrlinger, U	1997	No	+	+						+		+		3
Herrlinger, U	1998	No	+	+				+					+	3
Hoffman, G	2007	No	+	+				+				+		3
Huang, D	2010	No	+	+								+	+	4
Huang, Q	2007	No	+	+				+				+		3
Huang, Q	2010	No	+	+				+				+	+	2
Huang, S	2011	No	+	+				+						3
Huszthy, P	2006	No	+	+							+	+	+	3
Huszthy, P	2008	No	+	+								+	+	5
Huszthy, P	2010	Yes	+	+				+				+	+	3
Ikeda, K	2000	No	+	+	+			+	+		+	+	+	2
Ito, S	2010	No	+	+				+				+	+	3
Ito, S	2010	No	+	+						+				3
Iwadate, Y	2000	No	+		+			+	+		+			5
Iwadate, Y	2005	No	+	+	+			+				+	+	3
Jeong, M	2009	No	+	+				+				+	+	3
Jia, Q	2010	No	+	+	+			+	+	+		+	+	3
Kanai, R	2006	No	+	+				+				+	+	3
Kanai, R	2011	No	+	+								+	+	3
Kanai, R	2012	No	+	+				+				+	+	5
Kato, T	2010	No	+											3
Kikuchi, T	1999	No	+	+								+		3
Kim, C	2006	No	+	+				+			+	+		5
Kim, C	2007	No	+	+	+			+				+		3
Kim, S	2008	No	+	+						+		+	+	2

Name	***Year***	***TK model***	***1***	***2***	***3***	***4***	***5***	***6***	***7***	***8***	***9***	***10***	***11***	***Quality Score***
Kurozumi, K	2007	No	+	+				+				+	+	3
Kuwashima, N	2005	No	+	+				+				+		3
Lal, B	2005	No	+	+		+		+	+			+	+	5
Lamfers, M	2005	No	+	+				+				+	+	3
Lau, C	2009	No	+					+				+		2
Lee, E	2011	Yes	+	+								+		2
Lee, S	2012	No	+	+				+				+		3
Li, C	2007	No	+	+				+				+	+	3
Li, H	1999	No	+	+				+			+	+	+	4
Li, J	2011	No	+									+	+	1
Li, S	2005	Yes	+	+										2
Liang, C	2009	No	+	+				+				+		3
Liau, L	1998	No	+	+						+	+			4
Lichtor, T	1995	No	+					+						2
Lichtor, T	2002	No	+	+				+				+		3
Lichtor, T	2003	No	+	+										2
Liu, S	2012	No	+	+				+				+	+	3
Liu, Y	2002	No	+	+									+	2
Liu, Y	2011	No	+	+				+				+	+	3
Lu, W	2006	No	+	+							+	+	+	3
Lumniczky, K	2002	No	+	+				+				+		3
Lun, X	2006	No	+	+				+			+	+	+	4
Lun, X	2009	No	+	+	+			+				+	+	4
Ma, H	2002	No	+	+				+		+	+	+		5
Machein, M	1999	No	+	+				+					+	3
Maeda, M	2006	No	+	+							+	+		3
Maguire, C	2008	No	+	+								+	+	2
Manome, Y	1996	No	+	+				+		+	+	+		5
Marconi, P	2000	No	+	+				+				+		3
Markert, J	2012	No	+	+									+	2
Maron, A	1996	Yes	+					+					+	2
Matsuda, M	2009	No	+	+	+			+				+	+	4
Matsuda, M	2011	No	+	+									+	2
Meijer, D	2009	No	+	+							+	+	+	3
Mineta, T	1995	No	+	+				+		+		+	+	4
Miura, F	2002	Yes	+	+				+						3
Mori, K	2010	Yes	+	+										2
Mori, K	2010	Yes	+	+				+						3
Morioka, M	2002	No	+	+				+				+	+	3
Moriuchi, S	1998	No	+	+	+							+	+	3
Moriuchi, S	2000	No	+	+										2
Moriuchi, S	2005	No	+	+	+			+				+		4
Nafe, C	2003	Yes	+	+				+			+		+	4
Nam, M	1996	No	+					+				+	+	2

Name	***Year***	***TK model***	***1***	***2***	***3***	***4***	***5***	***6***	***7***	***8***	***9***	***10***	***11***	***Quality Score***
Namba, H	1998	No	+	+				+			+			4
Natsume, A	1999	No	+	+				+				+		3
Nestler, U	2004	No	+	+				+			+		+	4
Niranjan, A	2000	No	+	+				+						3
Ogren, S	2009	No	+					+						2
Ohlfest, J	2005	No	+	+				+				+	+	3
Okada, T	2001	Yes	+	+				+						3
Otsuki, A	2008	No	+	+								+		2
Paraskevakou, G	2007	No	+	+							+	+		3
Paul, S	2000	No	+	+	+			+				+		4
Pellegatta, S	2006	No	+					+				+		2
Peres, E	2011	No	+	+				+	+			+		4
Perri, S	2005	No	+	+				+				+		3
Persson, B	2010	No	+	+					+					3
Phuong, L	2003	No	+	+	+	+		+				+		5
Redaelli, M	2012	Yes	+	+				+				+		3
Roche, F	2010	No	+	+								+	+	2
Ross, J	1995	Yes	+					+						2
Ryu, C	2011	No	+	+				+				+	+	3
Saka, M	2010	No	+	+						+	+		+	4
Samoto, K	2002	No	+	+				+						3
Sandmair, A	2000	Yes	+	+				+						3
Santra, M	2010	No	+	+	+			+				+	+	4
Sato, H	2005	No	+	+				+				+	+	3
Schneider, T	2008	No	+	+	+			+		+	+	+		6
Shah, A	2006	No	+	+				+				+		3
Shah, A	2007	No	+	+								+		2
Shir, A	2006	No	+	+								+		2
Sonabend, A	2008	No	+	+	+			+				+	+	4
Sun, X	2012	No	+	+				+				+	+	3
Szatmari, T	2008	No	+	+				+				+		3
Tabatabai, G	2010	No	+	+						+	+	+	+	4
Tamura, K	2001	Yes	+	+	+					+			+	4
Tse, V	2004	No	+	+								+	+	2
Tseng, S	1997	No	+	+				+			+			4
Tsugawa, T	2004	No	+	+		+		+	+				+	5
Ueda, R	2008	No	+	+				+				+		3
Ulasov, I	2007	No	+	+					+			+	+	3
Ulasov, I	2007	No	+	+				+		+		+		4
Verheije, M	2009	No	+	+				+				+	+	3
Viita, H	2012	No	+	+				+				+	+	3
Vincent, A	1996	No	+	+				+			+	+	+	4
Vincent, A	1997	Yes	+	+	+			+	+	+	+	+		7
Visse, E	1999	No	+	+	+	+		+			+	+		6
Wang, R	1999	No	+	+								+	+	2

Name	***Year***	***TK model***	***1***	***2***	***3***	***4***	***5***	***6***	***7***	***8***	***9***	***10***	***11***	***Quality Score***
Wang, T	2001	No	+	+				+				+	+	3
Wang, Y	2011	No	+	+				+				+	+	3
Wei, M	1995	No	+	+				+		+				4
Wirth, T	2012	No	+	+				+				+	+	3
Wu, A	2007	No	+	+				+				+		3
Xu, D	2006	No	+					+				+		2
Yacoub, A	2003	No	+	+				+			+	+		4
Yacoub, A	2008	No	+	+							+	+	+	3
Yamanaka, R	1995	No	+	+				+		+		+		4
Yamanaka, R	1999	No	+	+				+				+		3
Yamanaka, R	2000	No	+									+		1
Yamanaka, R	2001	No	+					+		+	+			4
Yamanaka, R	2002	No	+	+							+	+		3
Yamini, B	2004	No	+	+		+		+	+			+	+	5
Yang, Y	2011	No	+	+				+				+	+	3
Yokoyama, T	2008	No	+	+	+			+	+			+		5
Yoo, J	2012	No	+	+				+				+	+	3
Yoshida, J	2002	No	+	+						+	+			4
Yoshikawa, K	2000	No	+	+				+				+	+	3
Yu, J	1997	No	+	+				+				+		3
Zang, H	2007	No	+	+				+						3
Zhang, W	2011	No	+	+								+	+	2
Zhang, Y	2002	No	+	+				+					+	3
Zhang, Y	2004	No	+	+		+		+	+			+		5
Zhang, Y	2004	No	+	+								+	+	2
Zhang, Z	2004	No	+	+										2
Zhu, H	2011	No	+									+		1
