# Supplementary material for: A systematic review and meta‐analysis of gene therapy in animal models of cerebral glioma: why did promise not translate to human therapy?
Source: Evid Based Preclin Med. 2015 Jan 20;1(1):e00006. doi: 10.1002/ebm2.6 (PMC5020579; doi:10.1002/ebm2.6)
Supplement: Supplementary file 5 — Appendix S5. Number of animals per group needed to achieve a set statistical power. [file EBM2-1-21-s005.pdf]

# *Supplementary material 5: Number of animals per group needed to achieve a set statistical power*

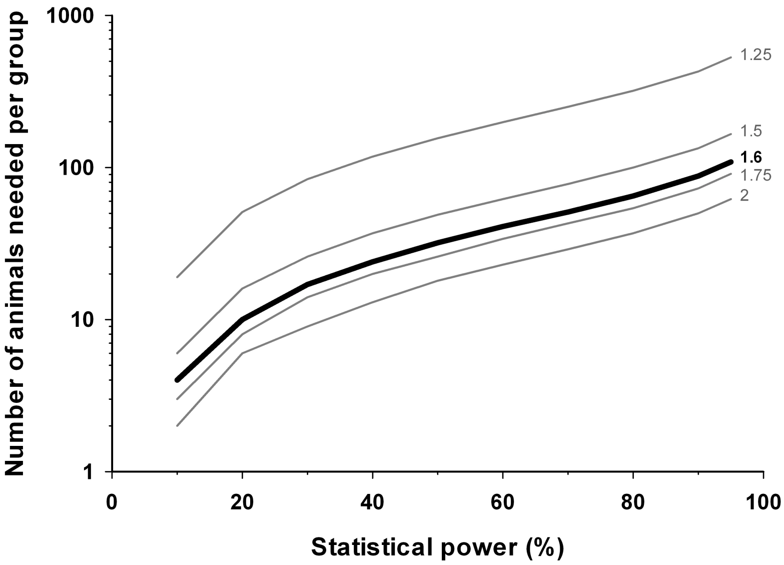


**Sample size estimation –** Number of animals needed per group to achieve a certain statistical power. The black line indicates values for the overall efficacy ascertained in this review (median survival ratio of 1.6); the grey lines highlight the same calculation for a range of median survival ratios that might be expected of a similar experiment (median survival ratios of 1.25, 1.5, 1.75 and 2, each line is labeled on the right hand side). The numbers inputted into this figure are tabulated below for convenience.

| Statistical power (%) | Median survival ratio | | | | |
|---|---|---|---|---|---|
| | *1.25* | *1.5* | *1.6* | *1.75* | *2* |
| *10* | 19 | 6 | 4 | 3 | 2 |
| *20* | 51 | 16 | 10 | 8 | 6 |
| *30* | 84 | 26 | 17 | 14 | 9 |
| *40* | 118 | 37 | 24 | 20 | 13 |
| *50* | 156 | 49 | 32 | 26 | 18 |
| *60* | 199 | 62 | 41 | 34 | 23 |
| *70* | 251 | 78 | 51 | 43 | 29 |
| *80* | 320 | 100 | 65 | 54 | 37 |
| *90* | 428 | 134 | 88 | 73 | 50 |
| *95* | 530 | 166 | 109 | 91 | 62 |
